# Heat and drought induced transcriptomic changes in barley varieties with contrasting stress response phenotypes

**DOI:** 10.3389/fpls.2022.1066421

**Published:** 2022-12-08

**Authors:** Ramamurthy Mahalingam, Naveen Duhan, Rakesh Kaundal, Andrei Smertenko, Taras Nazarov, Phil Bregitzer

**Affiliations:** ^1^ Cereal Crops Research Unit, USDA-ARS, Madison, WI, United States; ^2^ Department of Plant, Soils and Climate, Utah State University, Logan, UT, United States; ^3^ Institute of Biological Chemistry, Washington State University, Pullman, WA, United States; ^4^ National Small Grains Germplasm Research Facility, USDA-ARS, Aberdeen, ID, United States

**Keywords:** Barley, combined stress, drought, differential gene expression, gene networks, gene ontologies, heat, RNA-seq

## Abstract

Drought and heat stress substantially impact plant growth and productivity. When subjected to drought or heat stress, plants exhibit reduction in growth resulting in yield losses. The occurrence of these two stresses together intensifies their negative effects. Unraveling the molecular changes in response to combined abiotic stress is essential to breed climate-resilient crops. In this study, transcriptome profiles were compared between stress-tolerant (Otis), and stress-sensitive (Golden Promise) barley genotypes subjected to drought, heat, and combined heat and drought stress for five days during heading stage. The major differences that emerged from the transcriptome analysis were the overall number of differentially expressed genes was relatively higher in Golden Promise (GP) compared to Otis. The differential expression of more than 900 transcription factors in GP and Otis may aid this transcriptional reprogramming in response to abiotic stress. Secondly, combined heat and water deficit stress results in a unique and massive transcriptomic response that cannot be predicted from individual stress responses. Enrichment analyses of gene ontology terms revealed unique and stress type-specific adjustments of gene expression. Weighted Gene Co-expression Network Analysis identified genes associated with RNA metabolism and Hsp70 chaperone components as hub genes that can be useful for engineering tolerance to multiple abiotic stresses. Comparison of the transcriptomes of unstressed Otis and GP plants identified several genes associated with biosynthesis of antioxidants and osmolytes were higher in the former that maybe providing innate tolerance capabilities to effectively combat hostile conditions. Lines with different repertoire of innate tolerance mechanisms can be effectively leveraged in breeding programs for developing climate-resilient barley varieties with superior end-use traits.

## Introduction

Among the most consequential impacts of the ongoing global climate change, drought and high temperatures will adversely affect agricultural production world-wide (Fedoroff et al., 2010; Mahalingam et al., 2021). While singly occurring drought or heat stress can lead to yield reduction, the concomitant occurrence of these two abiotic stressors in field can be devastating (Barnabas et al., 2008; Awasthi et al., 2014; Cohen et al., 2021). Five or more recurring days of heat in which the daily maximum temperature is 5°C higher than the average maximum temperature is considered a heatwave (W.M.O, 2015). In the two decades spanning 1990-2010, in the US, combined heat waves and drought have increased compared to earlier decades (Mazdiyasni and AghaKouchak, 2015). Furthermore, climate models predict the intensity and frequency of such incidents will further increase (Lobell and Gourdji, 2012).

For breeding climate change resilient crop plants, a better understanding of the responses to combined drought and heat stress is important (Zandalinas et al., 2018). Responses to combined drought and heat stress in different crop species have previously been reviewed recently (Mahalingam et al., 2021). Combined heat and drought stress generate unique metabolic signatures in maize that are otherwise unaffected when the stressors are applied singly (Obata et al., 2015). In wheat lines subjected to combined heat and drought stress during pre-anthesis stage, proline content and number of tillers were identified as key attributes of tolerance to combined stress (Qaseem et al., 2019). In chick peas, the starch and sucrose content of seeds was significantly reduced in the combined heat and drought stress treatment during seed-filling stage when compared to singly applied stress (Awasthi et al., 2014). Apart from a reduction in yield in response to combined heat and drought stress, seed nitrogen was reported to be high and starch content was low in two Australian malting barley varieties (Savin and Nicolas, 1996). Combined heat and drought stress negatively impacted malting quality of US barley varieties (Mahalingam, 2017). These studies focused on the agronomic and physiological impacts of combined stress. There is gap in the knowledge about the molecular mechanisms of tolerance in plants operative during combined stress.

Photosynthesis machinery can recover once drought and heat stress is removed; however, flower, ovary or seed abortion are irreversible processes. Hence, drought and heat stress combination during the reproductive stages are more detrimental to crop yields (Barnabas et al., 2008; Mahalingam and Bregitzer, 2019). Drought and heat combination restricts the life cycle, decreases overall carbon assimilation and drastically shortens the grain filling period in crops (Awasthi et al., 2014). Meta-analysis of more than 120 case studies of heat and drought stress combination confirmed its negative impacts on harvest index, seed number and single seed size (Cohen et al., 2021). A key strategy to alleviate the influence of drought and heat stress on crop production and quality is identification of germplasm that can tolerate these stresses during post-anthesis and using them in breeding programs or identifying the genetic mechanisms of tolerance and moving those favorable genes/alleles into the current germplasm using modern biotechnological tools.

The main obstacle in the selection of genes conferring drought and heat tolerance is the complexity of plant responses to these types of stresses. Coping strategies to overcome abiotic stress like drought may be transient, such as reduced transpiration or hydrotropism, or entail developmental reprogramming such as deeper root system, reduction of leaf area or biochemical alterations such as osmotic adjustments to minimize water loss and improve water uptake (Hu and Xiong, 2014). Transient responses, developmental changes and biochemical modifications require a substantial rebuilding of plant metabolism and gene expression changes that keep changing with the onset and as the stress persists (Wiegmann et al., 2019). Insight into the complexity of plant response to combined stress can be appreciated *via* transcriptome profiling.

The majority of studies of drought induced transcriptome changes in barley focused on leaf tissue (Talame et al., 2007; Guo et al., 2009; Bedada et al., 2014; Wehner et al., 2016; Zeng et al., 2016) while few studies analyzed spikelets, awns, seeds (Abebe et al., 2010; Hubner et al., 2015), or crowns (Svoboda et al., 2016) and one examined leaves and roots (Janiak et al., 2018). Heat stress in different parts of the seeds were examined using microarrays (Mangelsen et al., 2011). Transcriptome changes in leaves and inflorescence in response to drought and combined heat and drought stress (Cantalapiedra et al., 2017) and proteomic alterations in young leaves subjected to heat, drought and combined stress have been reported (Ashoub et al., 2015).

In this study, drought, heat and combined heat and drought was imposed during heading stages in the tolerant barley variety Otis and a sensitive variety Golden Promise. Physiological traits were monitored during the stress regime and agronomic traits were compared at maturity. Transcriptomic differences associated with heat, drought, and combined heat and drought stress in these two contrasting lines were examined 1-day after initiating the stress and at the end of the 5-day treatment. RNA-Seq analysis revealed a greater number of differentially expressed genes in response to combined heat and drought stress compared to heat or drought stress in both GP and Otis. Interestingly, several genes with proven roles in abiotic stress tolerance such as trehalose biosynthesis, linolenic acid and glutathione metabolism were expressed at higher levels in Otis under non-stress conditions compared to Golden Promise. Identification of innately expressed genes with proven roles in abiotic stress tolerance in advanced breeding lines and modern varieties can accelerate the pace of climate-resilient cultivar development with superior end-use traits.

## Materials and methods

### Plant growth conditions

Seeds of Barley varieties Golden Promise (GP) and Otis were imbibed in water for three hours and three seeds were sown in each 2.5 L pots containing the potting mix as described earlier (Mahalingam and Bregitzer, 2019). Plants were maintained in the greenhouse till the first spikelet of the head had completely emerged corresponding to Zadok’s scale 5.9. This plant grow-out scheme was followed precisely for the three biological replications.

### Stress treatments

Pots with plants at heading stage were moved into growth chambers for heat stress and combined heat and drought stress experiments. The growth chambers were programmed to approximate the light intensity in the greenhouse (450 μmol m^−2^ s^−1^; 16 h of light and 8 h of darkness; 50% humidity). Plants were acclimated in the growth chamber for 48 h before the imposition of the stress treatments. The heat, drought and combined stress treatments were conducted as described previously (Mahalingam and Bregitzer, 2019), when the head on the main tiller became visible. During the heat stress, plants were manually irrigated with 550 ml of water, the same amount as control plants in the greenhouse under auto-irrigation. Plants were maintained in these stress conditions for five days. On the sixth day, the chamber was reprogrammed to simulate the conditions in the greenhouse. On day seven the plants were moved into the greenhouse until physiological maturity.

### Physiological measurements

Flag leaf and the leaf underneath the flag leaf from each plant were used for measuring the physiological traits with a Li-Cor 6400 Portable Photosynthesis system (Li-Cor, Lincoln, NE) as described previously (Mahalingam and Bregitzer, 2019). Stomatal conductance, net transpiration rates and net photosynthetic rates were recorded before the stress treatment and after the end of the stress treatments.

The leaf relative water content (LRWC) was calculated as described earlier (Schonfeld et al., 1988). LRWC% = (Fresh weight-Dry weight)/(Turgid weight-Dry weight) X 100. The LRWC measurements were conducted from the leaves sampled on the fifth day of the stress treatment.

For the physiological measurements with Li-Cor and the LRWC, two leaves were sampled from two different plants for each stress treatment and each genotype in each replication. Averages reported are based on data collected from at least five plants. The average measurements for the physiological traits of plants before the imposition of stress were compared with measurements from the same plants after five-days of stress treatment to determine statistically significant differences.

### Sample harvesting for RNA analysis

Flag leaf and heads were harvested from each plant and frozen immediately in liquid nitrogen. For each stress and corresponding controls, tissues were collected from five individual plants. Frozen tissue samples were wrapped in aluminum foils and stored in -80 ^∘^C. Tissues were collected one day into the stress treatment (early time point) and at the end of the five days of stress treatment (late time point).

### Agronomic traits

The weight of dry shoots and dry roots were recorded for each plant at maturity. The dry mature heads from each plant were collected in brown bags and threshed using a benchtop thresher (Model LT15; Haldrup, Poneto, IN). The seed weight was recorded for each plant and seed yield of five plants from each treatment were averaged.

### RNA isolations

Total RNA was isolated from the flag leaf and head tissues using the RNeasy plant mini kit (Qiagen). Two independent RNA isolations were done for each tissue from each of the biological replicates. Two genotypes, three different stress treatment (drought, heat, combined stress) and three corresponding non-stressed controls, two different tissue types (flag leaf and developing head), two time points (1 day into stress and 5^th^ day of stress treatment) equates to 48 RNA samples for one replication. Three replications of this entire experiment accounted for 144 RNA samples.

### Library construction and sequencing

QuantSeq method was used to generate 144 next-generation sequencing (NGS) libraries as described in the kit protocol at the University of Idaho sequencing facility.

### Read quality assessment and mapping to barley genome

The raw reads were trimmed to remove adapter sequences and ambiguous nucleotides and assessed for quality by running through several programs such as feature counts, STAR, Gene Counts, Cutadapt, SortMeRNA, and FastQC. The output from these various programs were summarized into a single report using MultiQC (version 1.8). The filtered reads were then mapped to the barley reference genome IBGS_version 3. The read mapping data were then combined with gene feature file to obtain the feature counts. This helped to identify uniquely mapped reads and reads that mapped to multiple genes. The uniquely mapped reads were used for identifying the differentially expressed genes (DEGs) by pair-wise comparisons to corresponding controls.

### Data analysis

Samples (libraries) were clustered in a multidimensional scaling plot (MDS plot) by the plotMDS function implemented in the Bioconductor package Limma in R (R Version 3.4.0, limma_3.32.2).

### Identification of DEGs

The gene expression levels were estimated using RNA-Seq by Expectation–Maximization (RSEM) (Li and Dewey, 2011). To perform differential expression analysis the DESeq2 R package (1.10.1) was used. This provides statistical routines based on the negative binomial distribution model for identifying differential expression. To control false discovery rate (FDR) the P-values were adjusted using the Benjamin and Hochberg’s approach (Benjamini and Hochberg, 1995). Both the q-value ≤ 0.05 and log_2_ (fold-change) ≥ 1 was set as the threshold for significant differential expression.

### Gene Ontology (GO) enrichment analysis of DEGs

GO analysis for biological process, cellular component, and molecular function of the DEGs was implemented by the GOseq R package. GO annotations for the entire barley genome determined based on the GOMAP strategy (Wimalanathan et al., 2018) was used as the reference for enrichment analysis using the Fisher’s Exact test and an FDR cutoff of 0.05. Genes within the enriched GOs from heat, drought and combined stress were compared for the leaf and head tissues separately.

### KEGG pathway enrichment analysis of DEGs

Statistical enrichment of genes in KEGG (http://www.genome.jp/kegg/) pathways was conducted using KOBAS software (Kanehisa and Goto, 2000; Mao et al., 2005). For the KEGG enrichment of the DEGs, a R package, ClusterProfiler was used (Yu et al., 2012).

### Weighted Gene Co-expression Network Analysis (WGCNA)

The raw reads counts were normalized using DESeq2 package and the signed co-expression network was created using WGCNA package (Langfelder and Horvath, 2008). The adjacency matrix was created by calculating the Pearson’s correlations between each gene. A value of nine was used as power parameter (β) on the scale-free topology requirement (Zhao et al., 2010). Then the adjacency matrix was used to calculate the topological overlap measure (TOM) and associated dissimilarity (1-TOM). Gene modules were then identified using a dynamic tree cutoff algorithm (minimum cluster size of 30, merging threshold function of 0.25) (Langfelder et al., 2008). Module membership (MM) was computed using Pearson correlations between expression levels and module eigengenes. A relatively high MM suggests that certain genes are well-connected inside the module.

### Module-traits relationships and functional categorization of modules

The module eigengenes (ME) was used to estimate the module-traits relationships by calculating the Pearson’s correlations between the ME and the traits of interest. Gene significance (GS) was used to correlate the trait of interest with the expression data of individual genes. The module-characteristics associations were calculated using the ME by computing Pearson’s correlations between the ME and the traits of interest. The modules were selected based on correlation >=0.05 and *p*-value <=0.05 in control vs treatments. Genes in the module were chosen when their intra-modular connection with that module was more than 0.2, and their intra-modular connectivity with all other modules was less than 0.2. The correlation between the gene’s expression profile and the ME’s expression profile was used to calculate intra-modular connectivity. Only the differentially expressed genes were extracted from each module and the network visualization was done using Cytoscape (Shannon et al., 2003). Using the pySeqRNA package (Duhan and Kaundal, 2020), the uniqueness of all the modules was determined based on gene ontology.

### Peroxisome abundance

Peroxisome abundance was measured using small fluorescent probe Nitro-BODIPY according to previously published procedure (Hickey et al., 2022). A 2-cm piece of the leaf was placed into 2 cm deep 96-well plate, immersed in a liquid nitrogen bath and ground with a tissue grinder (TissueLyser II, Qiagen, Venlo, Netherlands). The tissue powder was mixed with 0.8 ml of the extraction buffer A (EBA) containing 20 mM Tris HCl, pH7.4, 500 mM NaCl, 7M Urea, then the plate was rotated for one hour to extract total protein. The debris was removed by centrifugation at 3,000 *g* for 30 minutes and the supernatant was aspirated into a fresh plate. The reaction contained 20 μl of the extract, 80 μl of freshly prepared 2 μM solution of N-BODIPY, and 100 μl of water in 96-well plates and incubated for 10 min. The fluorescence intensity (490 nm excitation wavelength and 530 nm emission wavelength) was measured using Synergy Neo B spectrofluorometer (Biotek Instrument, Inc). Extracts from five individual plants (biological replicates), each three technical replicates, were measured per genotype and treatment. Two background values were measured per each 96-well plate: 20 μl of the protein extract in 180 μl of water; and 20 μl of 2 M N-BODIPY solution with 180 μl of water. These values were subtracted from the N-BODIPY fluorescence signal. The protein concentration was measured in each extract using the Bradford Reagent (Biorad Laboratories) with a calibration curve of known concentrations of Bovine Serum Albumin. The N-BODIPY fluorescence intensity was normalized by the protein concentration and calculated in arbitrary units per mg of protein.

## Results

### Growth and physiological responses to drought, heat, and combined stress

The stress experiments described in this study were conducted using barley plants that were in their heading stage. There were basic morphological differences between GP and Otis with the former bearing a lot more tillers during their vegetative growth phase. The leaves of Otis were broader, and the plants were taller than GP. Flag leaves of GP plants were smaller compared to the flag leaves of Otis.

**Figure 1:**
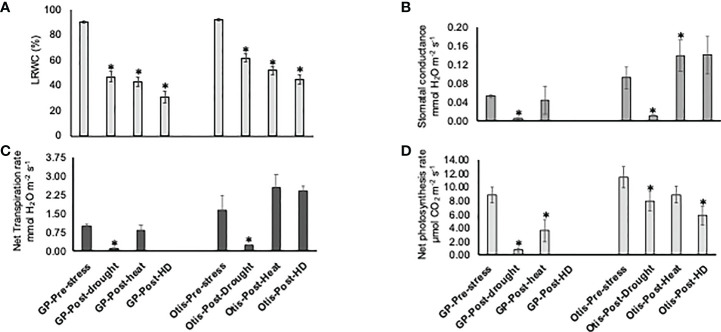
Physiological changes in Golden Promise and Otis under heat, drought and combined heat and drought stress **(A)** Leaf Realtive Water Content (LRWC). **(B)** Stomatal conductance. **(C)** Net transpiration rate. **(D)** Net photosynthetic rate. Values are the means of five plants. Bars represent standard errors of the means. *P < 0.05.

Leaf relative water content (LRWC) was reduced in both the lines in response to heat, drought, and combined stresses. Overall, the reduction in the LRWC was greater in GP leaves compared to Otis (**Figure 1A**). Pre-stress stomatal conductance (SC) showed significant difference between the two varieties, with Otis registering values that were nearly 50% higher than GP (**Figure 1B**). In response to drought, both varieties showed significant reduction in their SC. On the contrary, heat and combined stress increased the SC of leaf by nearly 45% in Otis. Interestingly SC of GP leaf subjected to heat stress were like that of control leaves. Patterns of changes in net transpiration rates were identical to the pattens observed for SC in both varieties (**Figure 1C**). Net photosynthesis rates declined by nearly 50% in response to heat and 84% in response to drought in GP (**Figure 1D**). Leaves of Otis showed a 25% reduction in photosynthesis in response to heat, a 32% decrease in response to drought and 50% reduction in response to combined stress. In the GP plants the impact of the combined stress on the leaf was too severe and did not provide reliable measurements for SC, net photosynthesis, and transpiration rates.

### Agronomic impact of drought, heat, and combined stress

**Figure 2:**
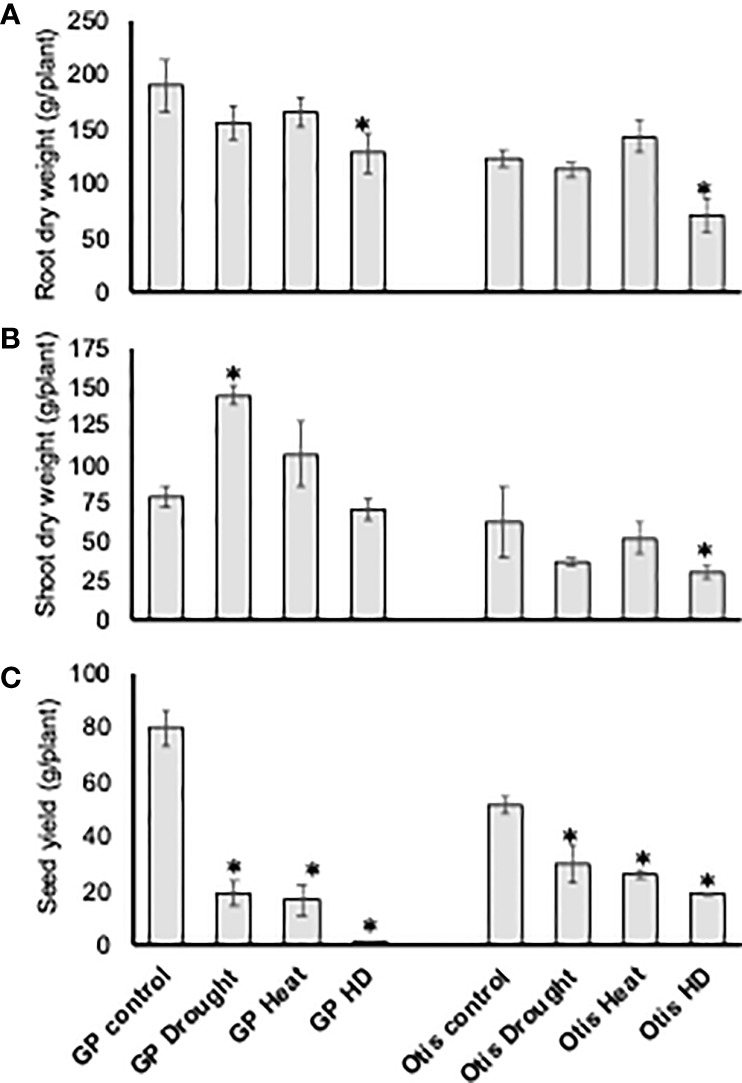
Analysis of dry root biomass **(A)**, shoot biomass **(B)**, and seed yield **(C)** in response to heat, drought and combined stress in Golden Promise and Otis. Error bars represent standard deviations of the mean of five observations from five different plants for each treatment (n = 5). Statistically significant differences in measured traits when compared to non-stressed control plants are denoted by ‘*’ above the bars (*P-value <0.05).

In both varieties there was no significant difference in the root biomass in response to singly applied heat or drought stress. However, in response to combined stress both lines showed a nearly 40% reduction in their root biomass (**Figure 2A**). In GP the shoot biomass doubled in response to drought stress and by 30% in response to heat stress. Interestingly, combined stress did not cause any significant change in GP (**Figure 2B**). On the contrary, in Otis, the shoot biomass decreased by more than 40% in response to combined stress and a similar decreasing trend was observed in response to singly applied heat or drought stress but was not statistically significant.

Given the profuse tillering habit of GP the seed yield per plant was 60% higher compared to Otis plants under control conditions (**Figure 2C**). However, the seed yields of GP were reduced by 75% in response to heat or drought and by nearly 95% in response to combined stress. In Otis the reduction in yield per plant was about 40% for the singly applied stress and about 60% for the combined stress. Based on the seed yield data, Otis was nearly 50% and 300% higher yielding compared to GP in response to singly applied stresses and combined stress, respectively.

**Figure 3:**
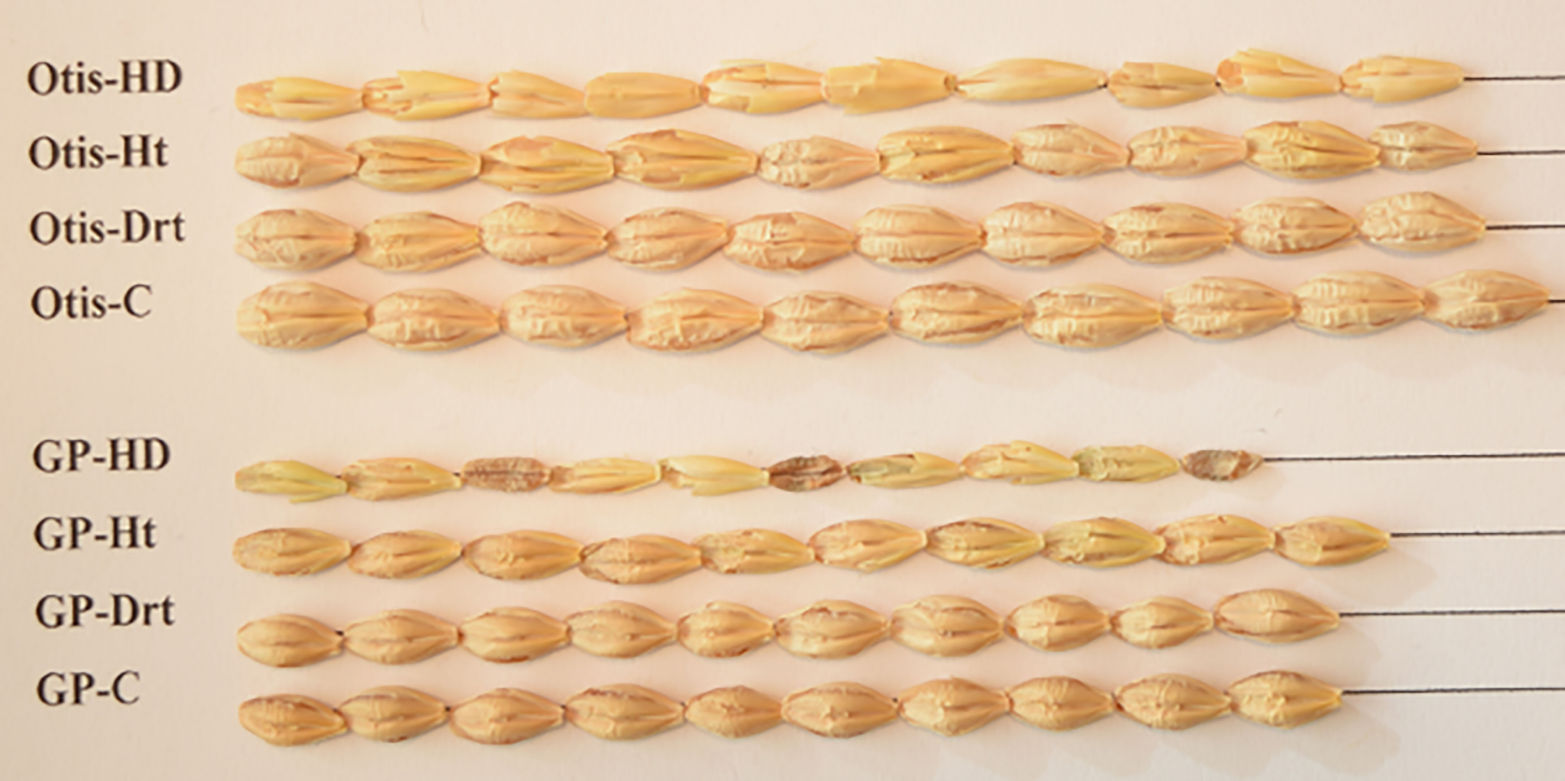
Seed physical characteristics of Golden Promise (GP) and Otis collected from plants subjected to heat (Ht), drought (Drt), combined heat and drought stress (HD) and control (C). Ten seeds randomly sampled from the seed bags were placed on a paper and arranged on a straight line and photographed using a Nikon camera.

The average length of Otis seeds was nearly 18% longer than the GP seeds under control conditions (**Figure 3**). Interestingly, heat stress increased the average seed length of GP seeds. Though the average seed length of GP and Otis seeds were reduced in response to combined stress, there was significant variation. GP seeds did not show any significant change in seed length in response to drought stress. An overall tendency towards reduction in seed length in response to stress was seen in Otis but was not statistically significant. The reduction in seed width was more obvious in Otis seeds subjected to heat stress (~23%) compared to GP (~11%). In response to combined stress, both varieties showed a reduction of more than 30% in seed width. GP seeds showed a slight increase in seed width in response to drought while Otis seeds showed a slight decrease in width. However, these differences were not statistically significant.

### Mapping of the RNA-seq reads to the barley genome

After quality trimming, an average of 79.4% of the sequences per library in Golden Promise and 78.4% of sequences from Otis were aligned to the barley reference genome (**Supplementary Table 1**). More than 96% of reads were mapped to unique genes in the barley genome.

### Overview of the relationships among the RNA-seq samples

**Figure 4:**
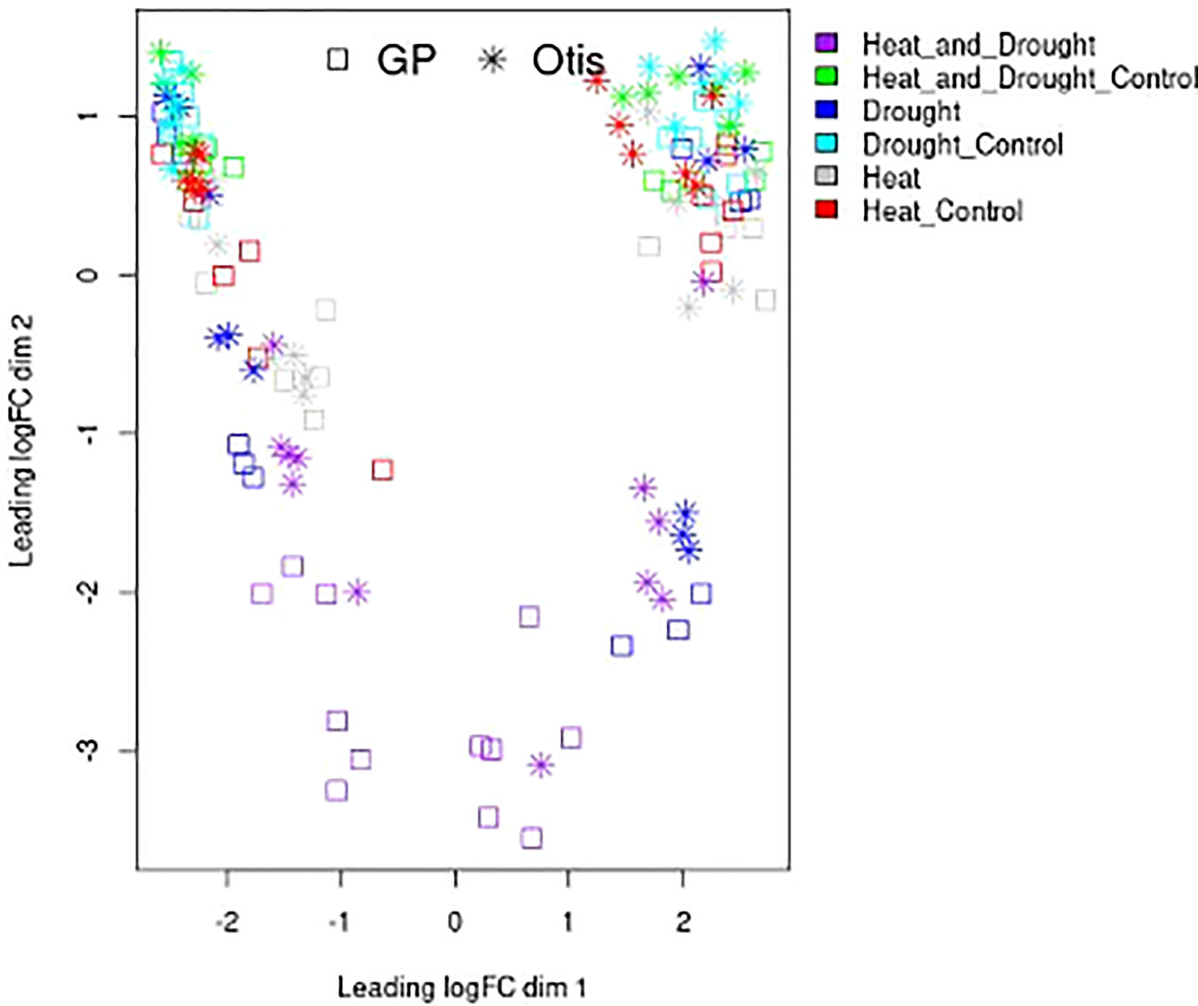
Multidimensional scaling plot of replicated RNA-Seq samples. Features on the plot represent libraries of control, drought, heat and combined stress treatments from flag leaves and head tissues collected after 1 day and 5 days of treatment. Spatial arrangement of the various RNA-seq libraries is based on their calculated distances estimated using root-mean-square deviation for the top 500 genes with the largest standard deviation among all samples. The square represents GP libraries while the * represent Otis libraries. The color code for the heat, drought and combined heat and drought and corresponding controls is shown on the right.

Transcriptomic relationships between the type of stress and tissue sample were determined in a multidimensional scaling (MDS) plot (**Figure 4**). Resulting distances between control and treatment samples are displayed as the leading log2-fold change (i.e., estimated root-mean-square deviation for the top 500 genes with the largest standard deviation among all samples). This analysis visually displays relationships among samples (stress treatment and tissue types) based on their spatial arrangement. The clustering of the control samples in the top left and top right was expected and represent the diversity in the biological material used for the analysis - flag leaf and head tissues, grouped near the left and the right side, respectively. Combined stress samples showed the most significant separation. The later time point of the drought stress showed a significant separation compared to the early time point. Heat stressed samples seemed to show the least separation and were found in closer proximity to the controls.

### Differentially Expressed Genes (DEGs) in response to heat, drought, and combined stress

**Figure 5:**
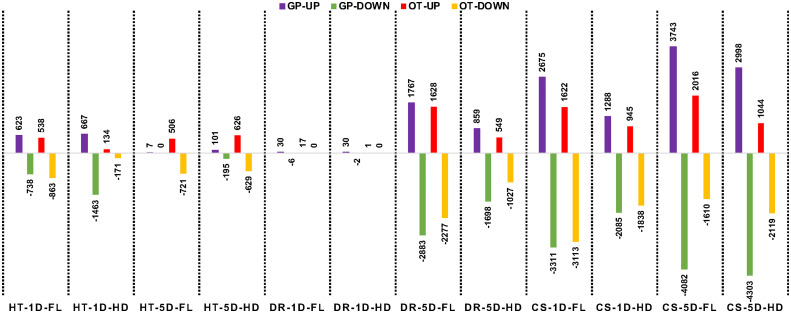
Number of differentially expressed genes in the flag leaf (FL) and head (HD) tissues at 1-day after stress (1D) or 5 days after stress (5D) in Golden Promise (GP) and Otis (OT) observed in response to heat (HT), drought (D), and combined stress (CS). Upward pointing bars represent upregulated genes and the downward facing bars represent the down regulated genes. Normalized counts for a gene from a stressed sample library is divided by the corresponding normalized counts for the same gene from the matched control sample library. A log_2_ > 1.0 and FDR of 0.05 was used as the cutoff to call a gene as being differentially expressed.

For each of the stress treatments, comparisons were made to time-matched non-stress controls for both the flag leaf and head tissues. A log2-fold cutoff ≥ 1 and an FDR of 5% were used as the defining criteria for DEGs. Of the 24 comparisons in this study (**Supplementary Table 2**), the lowest number of DEGs was in 1-day drought samples in both lines and in both tissues (**Figure 5** and **Supplementary Figure 1**). However, by the end of the fifth day of stress 4650 genes were differentially expressed in flag leaves of GP and 3905 in Otis, while the corresponding numbers in head tissue were 2467 and 1576, respectively (**Figure 5**).

In response to heat stress, both GP and Otis showed some marked differences in their tissue-specific and temporal transcriptional responses. In GP, more genes were differentially expressed in head compared to flag leaf at the early time point. On the contrary, in Otis nearly three-fold more genes were differentially expressed in the leaf compared to the heads at this timepoint. In the 5-day heat-stressed samples, the number of DEGs in the head and leaf tissue of Otis were similar. The very low number of DEGs observed in GP could be due to the severe heat stress damage incurred to flag leaf and the developing heads. Despite the minimal response to drought at one day, the strong response evoked by 5-days of drought was significantly higher than the transcriptional responses evoked after 5-days of heat stress.

Combined heat and drought stress caused massive changes in the transcriptomes of both these varieties. Within 1-day of combined stress, more than 9300 genes were differentially expressed in GP and 7500 genes in Otis and the number of genes differentially expressed genes was greater in the leaf tissue compared to heads. By five days of combined stress, the number of differentially expressed genes increases to more than 15,000 genes in GP while these numbers were nearly 50% less (6789) in Otis. These results clearly showed massive transcriptional changes in response to combined stress, in both genotypes and tissues when compared to heat or drought stress. Furthermore, the transcriptional changes observed in the sensitive GP was significantly larger compared to the tolerant Otis.

### Comparing DEGs responsive to drought, heat, and combined stress

**Figure 6:**
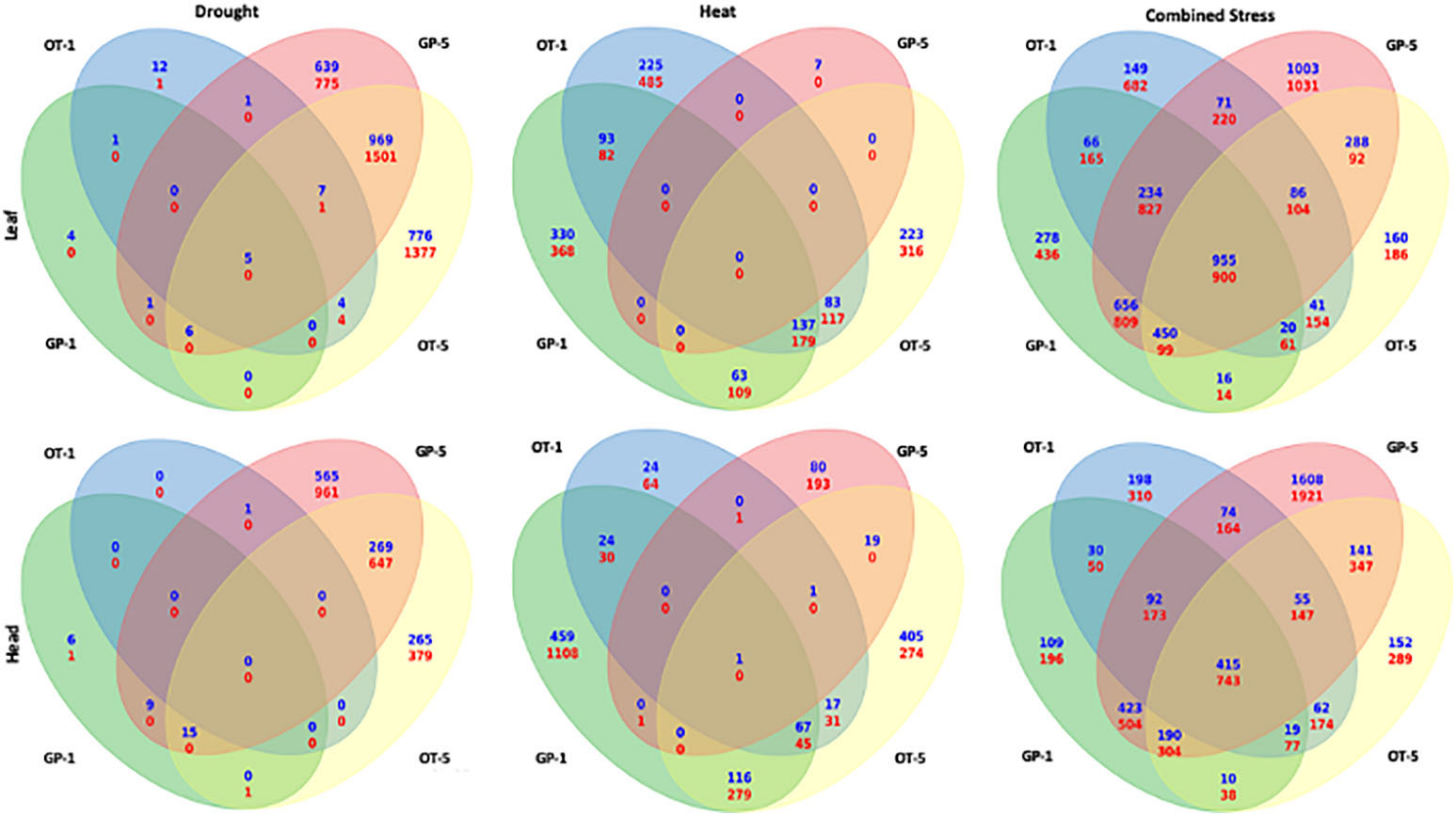
Venn diagram showing the overlap between DEGs responsive to drought (left), heat (middle), and combined stress (right). The top panel shows the comparisons from the leaf libraries and the bottom panel shows the comparisons from head libraries. In each Venn diagram the left side ovals represents the 1-day tissue sample and the right-side ovals represent the 5-day tissue sample.

The number of differentially expressed genes that were common for the early (1-day) and later stages (5 days) of singly applied heat stress in GP and drought stress in Otis did not show any overlap (**Figure 6**). This observation was consistent for flag leaf and head tissues. Thirty genes in the flag leaf and 55 genes in the head tissues showed an overlap between 1 and 5-days of drought in GP, while 414 genes in leaf and 196 genes in head tissues overlapped between the 1 and 5-days of heat stress in Otis (**Figure 6**). On the contrary, there were significantly more genes that were common to 1 and 5 days in response to combined stress when compared to singly applied stresses. Furthermore, significant differences were observed in the pattern of overlap in the two varieties. In flag leaf of GP more than 4400 differentially expressed genes were common between 1 and 5 days, while in Otis around 1000 genes were found to overlap between the two time points. Consistent with this observation, the number of uniquely differentially expressed genes at early stage in GP was 470 genes, while in Otis this was close to 2450 genes. This pattern was reversed at the 5-day time point with GP recording 2222 genes while in Otis 660 genes were uniquely differentially expressed. In the head tissue, GP had 2600 commonly differentially expressed genes between 1 and 5 days compared to 1750 genes in Otis. Like the pattern in the flag leaf, in GP heads 3416 genes were uniquely expressed at the 5-day time point compared to 360 genes observed at 1 day. In Otis head tissues, approximately about 600 genes were unique for the 1-day and 400 at the 5-day time point. Based on these observations the transcriptional response to counter combined stress in Otis appears to be regulated and consistent while GP appears to unleash a massive reprogramming especially during the later time point.

### Gene Ontology (GO) enrichment analysis of DEGs

**Figure 7:**
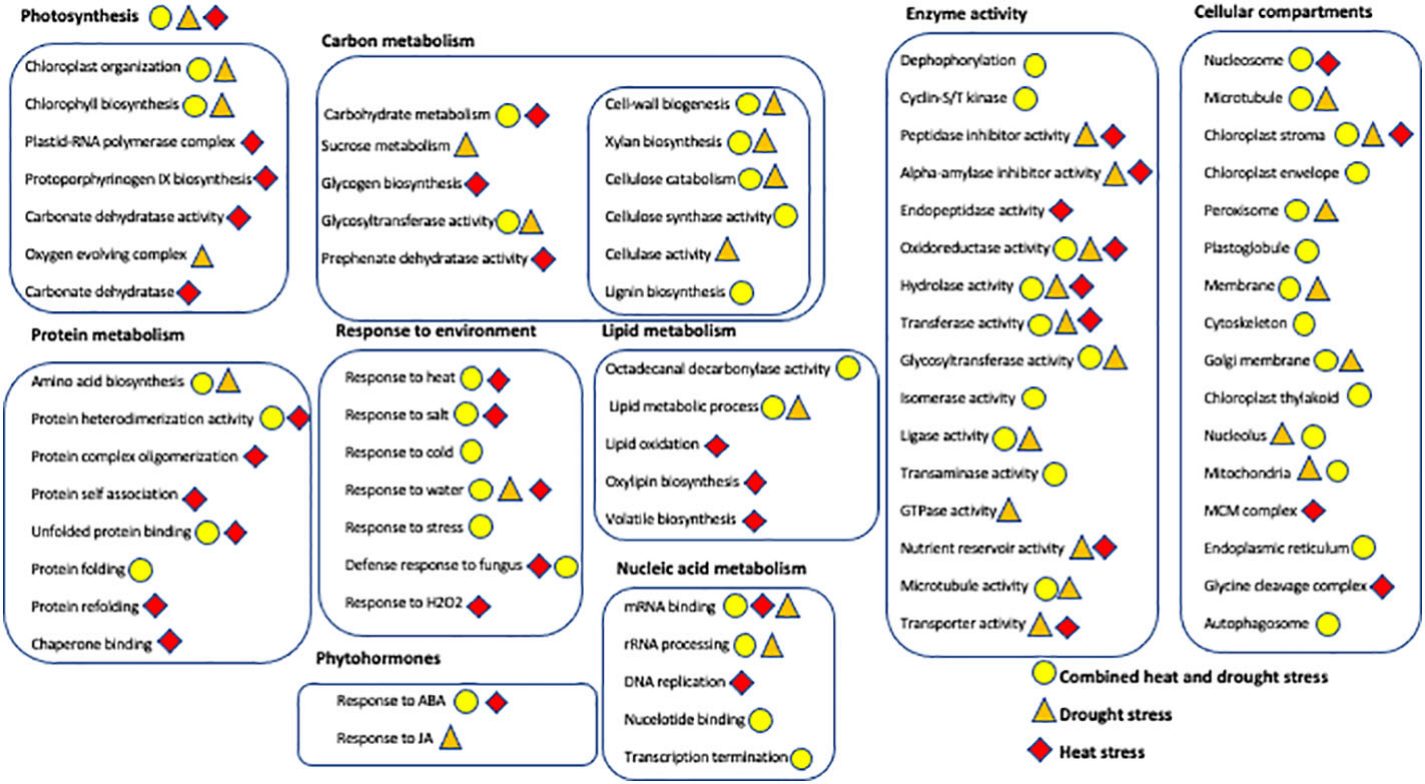
Overview of the Gene Ontology enrichment analysis showing the major biological processes, molecular functions, and cellular compartments of differentially expressed genes from leaves and head of Golden Promise and Otis in response to drought (brown triangles), heat (red diamond) and combined stress (yellow circle). Metabolic processes, molecular functions and genes encoding for proteins with specific activities are grouped within boxes. Only statistically significant GOs (P-value <0.001 and FDR<0.05) and those containing three or more DEGs were used for creating this illustration.

The GO terms with an FDR cutoff of 0.05 containing five or more genes were identified for further analysis and are presented in **Figure 7** (**Supplementary Table 3**). GOs wherein most genes that were differentially expressed were either in GP or Otis are described below for each of the three stresses.

**Figure 8:**
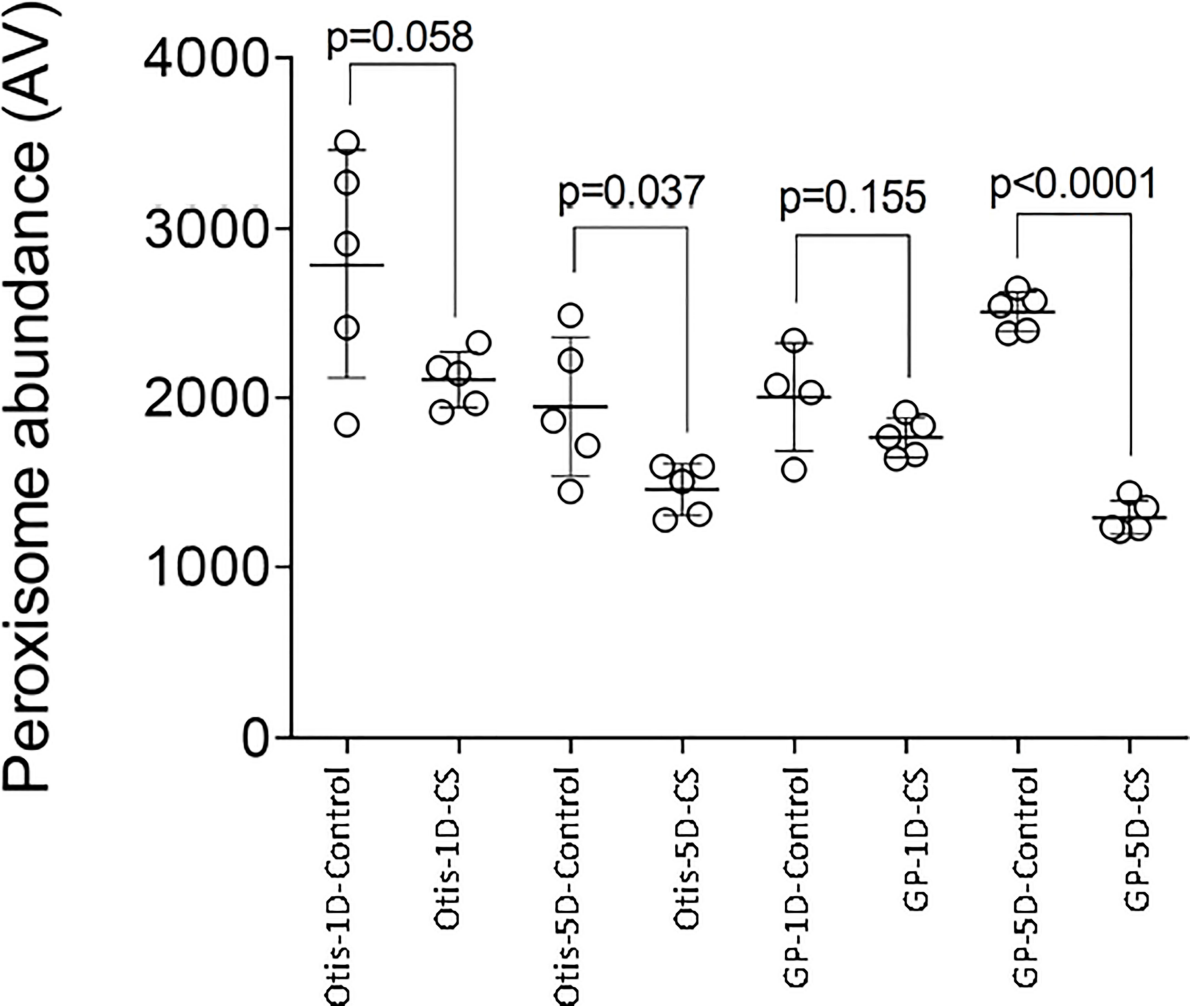
Impact of combined heat and drought stress (CS) on peroxisome abundance in Golden Promise (GP) and Otis. Values are from five different samples. P-values represent the pair-wise comparison of control and stressed leaf samples for each time point and genotype.

Combined stress: Peroxisomal genes were enriched in the DEGs in response to combined heat and drought stress in leaves of both GP and Otis at the early time point (**Supplementary Table 4**). This enrichment was observed even at the later time point in GP in response to combined stress and drought alone. Among the differentially expressed genes associated with peroxisomes, 25 genes were up and 15 were down in GP, while 22 were up and 9 down in Otis. At the end of the 5-day stress regime this pattern was maintained with 32 genes being up and 14 down in GP. One catalase gene was down only in GP while another catalase gene was induced in both genotypes at the early time point. At the 5-day time point two catalases were down in GP and one was induced in both the genotypes. One of the genes annotated as PEX11 was down in both GP and Otis while two other PEX11 genes were upregulated in both genotypes at 1- and 5-day after stress. It was observed that the extent of downregulation of the catalases and peroxin genes were stronger in GP compared to Otis. In the 5-day drought leaf sample, three PEX11 genes were identified, of which two were up in both GP and Otis while one was down only in the former. The extent of differential expression was stronger in GP than in Otis. Intriguingly, quantification of peroxisomes in the leaves showed a larger reduction in peroxisome abundance in GP compared to Otis at 5-days of combined stress (**Figure 8**).

Autophagy genes were strongly enriched in the DEGs in the 5-day leaf samples of GP. Of the 19 genes that were identified as being associated with this GO, six genes had annotations indicating they were ATG family genes. In particular, ATG8 which is a marker gene for peroxisome degradation was found to be induced strongly in GP compared to Otis. This suggests that GP may be experiencing higher levels of oxidative stress that leads to oxidation of proteins and turnover of organelles that trigger the autophagic flux. This is further supported by the enrichment of the GO for unfolded protein binding in the stress sensitive GP.

In Otis, the GO for oxido-reductase activity is enriched in the set of DEGs (**Supplementary Table 4**). Of the 241 genes associated with this category there were 74 genes that were differentially expressed only in Otis. Noteworthy genes among the induced ones were a gene involved in proline biosynthesis, several peroxidases, dioxygenases, flavin monooxygenase, and genes in the GABA shunt pathway. Among the repressed genes were Rubisco, GA20 Oxidase, and chlorophyll biosynthesis genes.

In the head tissue of GP, combined stress evoked differential expression of six different genes annotated as cyclin-dependent protein ser/thr protein kinase inhibitors of which one gene was induced and five genes were repressed.

In the head tissue of Otis plants, the GO xylan biosynthesis was enriched. Analysis of the 14 DEGs associated with this GO showed that seven were repressed only in Otis. The remaining seven genes were repressed in both GP and Otis, but the extent of repression was stronger in the latter.

Similar patterns of stronger repression of eight genes associated with GO for cellulose catabolic process was observed in Otis. Among the repressed genes were three endoglucanases that were strongly downregulated only in Otis. In GP heads the GO for cellulose synthase (UDP-forming) activity was enriched and again 11 of the 12 genes were repressed. Among the repressed genes, four were only identified in GP. Interestingly, this GO was identified in the heads of Otis at the 5-day time point. Among the 13 DEGs associated with this GO, 12 were repressed and one was induced in Otis and none of these genes were differentially expressed in the heads of GP.

Drought stress: The GO for peroxisomes was enriched in response to drought in GP leaves (**Supplementary Table 5**). Of the 29 DEGs associated with this GO, nine were repressed and 20 were induced. Notable among the induced genes that showed significant upregulation in GP compared to Otis were two genes – urate oxidase and acyl-CoA oxidase whose activities can lead to generation of hydrogen peroxide.

Among the 70 genes associated with the GO mRNA binding, 64 were repressed in GP and six were induced. Many of these genes were associated with rRNA processing, and proteins associated with forming larger complexes such as WD-40 repeat containing proteins, tetra and pentatricopeptide repeat containing proteins.

In Otis, there were 166 genes associated with the transmembrane transport process. Of these 87 were repressed and 79 were induced. Among the induced genes that were unique to Otis several were annotated as ABCG type transporter that are implicated in hormonal transport especially ABA. Interestingly there were two sugar transporters that were unique to Otis but showed opposites patterns of expression.

The octadecanal decarbonylase activity was enriched among genes differentially expressed in response to drought in Otis. Of the 11 genes associated with this GO, eight were induced and three were repressed. Two genes that were uniquely induced only in Otis, *Glossy1* and *Eceriferum1* homologs have been known to be involved in the biosynthesis of long chain fatty acids leading to enhanced wax production and rendering plants tolerant to drought.

The oxygen evolving complex (OEC) was an enriched GO cellular compartment in response to drought in Otis. Subunits of all three major genes (psbO, psbP and psbQ) of the OEC were identified and all these genes were repressed in both Otis and GP. Two subunit genes of the psbP domain important for binding of the chloride and calcium ions and making them available to PSII was downregulated only in Otis.

One of the most interesting GO terms that was identified in Otis heads was the negative regulation of endopeptidase activity. All the 18 genes associated with this GO were strongly induced in Otis and more importantly 14 of these genes were only observed in Otis but not in GP. Twelve of these were annotated as the serine type endopeptidase inhibitors, five were cysteine endopeptidase inhibitors and one gene contained the soybean trypsin inhibitor domain.

Sucrose metabolism was enriched in Otis heads. Six genes were associated with this GO of which four were induced and two were repressed in Otis in response to drought. Of the three genes that were uniquely induced only in Otis, two were annotated as sucrose synthase and one was a sucrose symporter.

In GP heads, the GO for response to JA was significant and included seven genes of which five were induced and two were repressed. Among induced genes were three transcription factors- two ERFs and a WRKY type TF, ornithine aminotransferase, important for proline biosynthesis, and an Inositol-polyphosphate phosphatase that can be crucial for stress signaling.

Heat stress: The GO for transmembrane transport was enriched in GP and comprised of a set of 76 genes of which 15 were induced and 61 were down regulated (**Supplementary Table 6**). Only two of the GP-induced genes associated with this GO was identified in Otis and one of them encoding a major intrinsic protein showed opposite pattern of expression. Of the 61 down regulated genes, 50 were unique to GP and only 11 genes were identified in Otis and showed similar changes in their expression in response to heat.

The GO for lipid transport was enriched in Otis and included a set of 15 genes of which 14 were down regulated. Only three of the genes associated with this GO was identified as differentially expressed in GP. Interestingly, all the genes associated with this GO were annotated as the non-specific lipid transfer proteins (nsLTPs). In the 5-day leaves of Otis, there were six genes associated with the zinc ion transport and all of them were down regulated.

In GP heads the ABA response GO was enriched one day after heat stress. Of the 17 genes identified as differentially expressed 11 were induced and six were repressed. Notable among the induced were the LEA, dehydrin8 and HVA22 genes. Among the repressed genes was a bZIP46-like TF that includes the well-known ABI5 gene.

Carbonate dehydratase activity was enriched in Otis heads. Of the seven genes associated with this GO, two were induced while five were strongly down regulated. The differential regulation of the members of this gene family only in Otis suggests a role for these genes in the heat tolerance trait. Since these genes are affected strongly by the zinc, it is possible that the observed differential expression could be indirectly mediated by zinc, wherein we observed a strong downregulation of zinc transporter genes only in Otis.

The GO for negative regulation of peptidase activity was enriched in the leaves of GP at the 1- day time point and in the heads of Otis at the end of treatment. Of the 20 genes associated with this GO in GP, 6 were up regulated, 14 were downregulated and none of these genes were identified in Otis. Of the 14 genes associated with this GO in Otis heads, 13 were strongly induced and one was down regulated. Only two of these genes were identified in GP and interestingly, these two genes showed opposite patterns of expression when compared to Otis. These two genes (HORVU.MOREX.R3.2HG0122620 and HORVU.MOREX.R3.3HG0309490) were down regulated in GP heads. As described earlier this GO was also identified in response to drought stress in Otis and hence, we compared the genes associated with this GO in the two stresses. Nine genes associated with this GO were common between heat and drought stress and eight of these showed similar patterns of gene expression in the head tissue. One gene (HORVU.MOREX.R3.3HG0309490) that was strongly induced in GP in response to drought was down regulated in heat stress.

### Weighted gene co-expression network analysis

**Figure 9:**
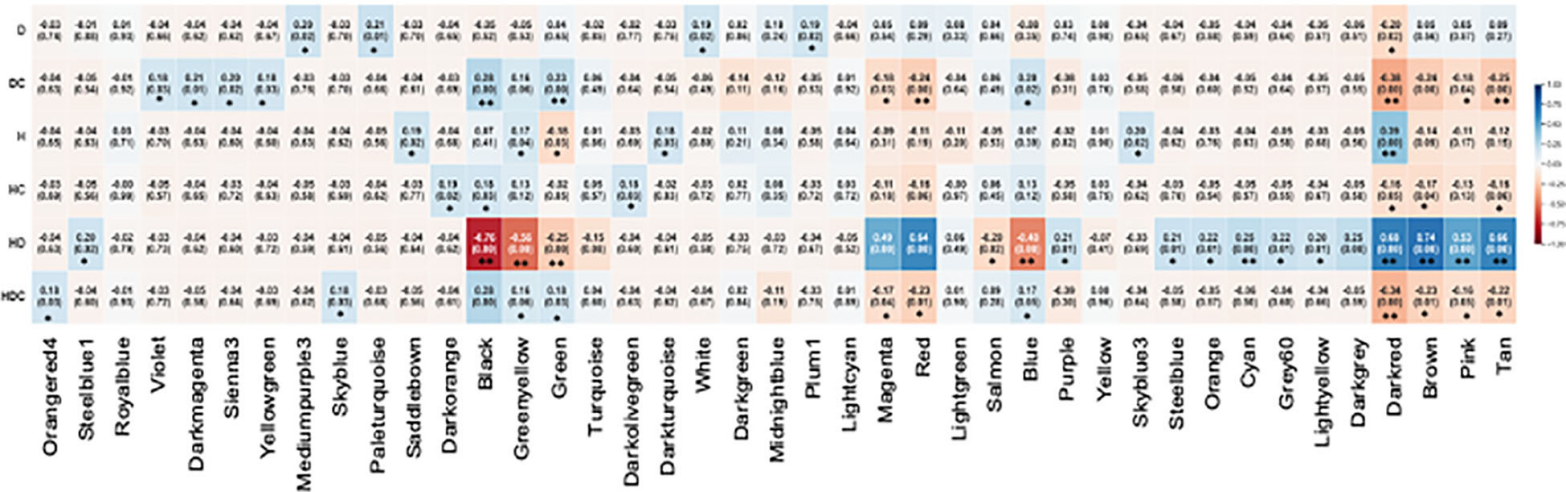
Matrix showing Module-Trait Relationships (MTRs) of different WGCNA modules under drought (D), heat (H) and combined heat and drought stress (HD) and the corresponding control modules for drought (DC), heat (HC) and combined heat and drought (HDC). The colors represent the various modules (**Supplementary Figure 2**). The numbers represent Pearson correlation coefficients, and the p-values are in parenthesis. Positive correlation is colored in blue while negative correlation is colored in red. Statistically significant correlations are indicated by *p-value <0.05 and >0.01, **p-values <0.01.

The WGCNA analysis clustered the genes identified in this study into 41 modules (**Supplementary Figure 2**). The module-trait specifications using Pearson correlation to link modules to stresses identified 55 significant correlations (**Figure 9**). The largest number of significant correlations (18) were associated with the combined stress. In the individually applied heat or drought stress, five significant correlations were identified. For the construction of gene networks, two selection parameters were considered. The first criterion was the number of genes in the module. Of the 41 modules, 19 contained less than 100 genes, 12 had more than 100 but less than 1000 and 3 had more than 1000 genes (**Supplementary Figure 3**). Secondly, we focused on those modules which showed opposite patterns of correlations between the stress and corresponding stress modules. Nine modules passed these criteria. The darkred module was identified in heat, drought, and combined stress. Black, green, magenta, red, blue, brown, pink, and tan modules were associated with the combined stress but not the singly applied stresses.

**Figure 10:**
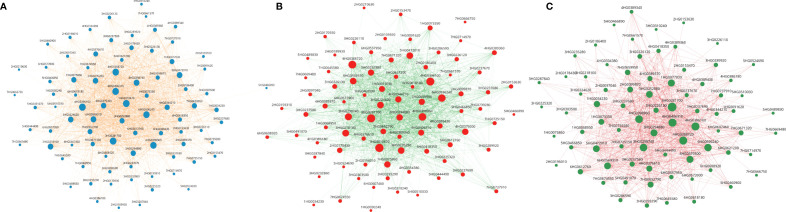
**(A)** Gene networks for 102 DEGs involved under heat and drought stress in the Dark red module (108 genes). Size of the node represent the degree. All genes’ nodes are represented in blue color while edges are represented by tan color. **(B)** Gene networks for 87 DEGs in response to heat stress in the Dark red module (108 genes). Size of the node represent the degree. All genes are represented in tomato color while edges are represented by green color. **(C)** Gene networks for 42 DEGs involved under drought stress in the Dark red module (108 genes). Size of the node represent the degree. All genes are represented in green color while edges are represented by pink color.

In the darkred module with 108 genes, networks comprising 101, 87 and 48 genes were identified among differentially expressed genes in combined stress (**Figure 10A**), heat (**Figure 10B**) and drought stresses (**Figure 10C**) respectively. Of the 48 genes in the drought network, 44 were also identified in the heat and combined stress networks. Between the heat and combined stress networks there were 42 common genes.

**Figure 11:**
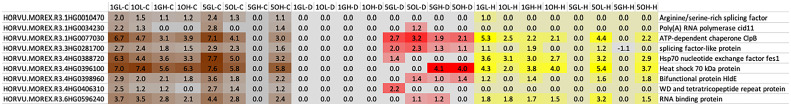
The nine hub genes identified in the darkred module for heat, drought, and combined stress in barley. Hub genes were selected based on the topological coefficients (<0.4) and closeness-connectivity values (>0.8). The matrix shows the differential expression values for each time point (1 or 5), genotype (Golden Promise-G and Otis-O), tissue type (L-Leaf; H-Head) and stress (D: Drought; H: Heat; C: Combined stress). The intensity of the color within each (brown: combined stress; Yellow: Heat; Red: Drought) indicates the extent of differential expression of the hub genes. The left column shows the gene identifiers, and the right column shows the IPK gene descriptions.

The network analysis indicated there were six hub genes in the heat, drought and the combined stress networks based on clustering and topological coefficient (**Supplementary Table 7**). Three were identified as hub genes in all three stress conditions, three were identified in two stress states (heat and combined stress or drought and combined stress), two genes were associated only with drought and one hub gene was associated only with heat stress. Four genes associated with RNA metabolism were identified as hub genes and included two genes associated with alternate splicing, a polyA polymerase and an RNA binding protein of unknown function. HSP family chaperones such as HsP70, ClpB and Hsp70-dependent nucleotide exchange factor were identified as the hub genes in these networks associated with heat, drought, and combined stress (**Figure 11**).

### Transcription Factors (TFs) responsive to heat, drought, and combined stress

Between the two varieties there were more than 1000 genes encoding TFs that were differentially expressed and consistent with the patterns observed for the whole DEG data set (**Supplementary Table 8**). Combined stress caused extensive up-regulation (232 in GP and 130 in Otis after 1 day; 179 in GP and 193 in Otis at 5 days) as well as down-regulation of TFs (269 in GP and 250 in Otis after 1 day; 226 in GP and 218 in Otis after 5 days). One day after drought stress evoked the lowest number of TFs in both lines. In contrast to drought stress, 1 day of heat stress caused upregulation of 71 TFs and downregulation of 110 TFs in GP while 50 were up and 48 were down in Otis. However, 5 days after drought stress, 181 TFs were up in GP and 150 in Otis, while 229 in GP and 177 in Otis were down regulated. In the 5-day heat stress samples the leaf tissues from GP were severely damaged and in the head tissue only two were up regulated and 26 were down, while 52 were up and 82 were down in Otis heads.

**Figure 12:**
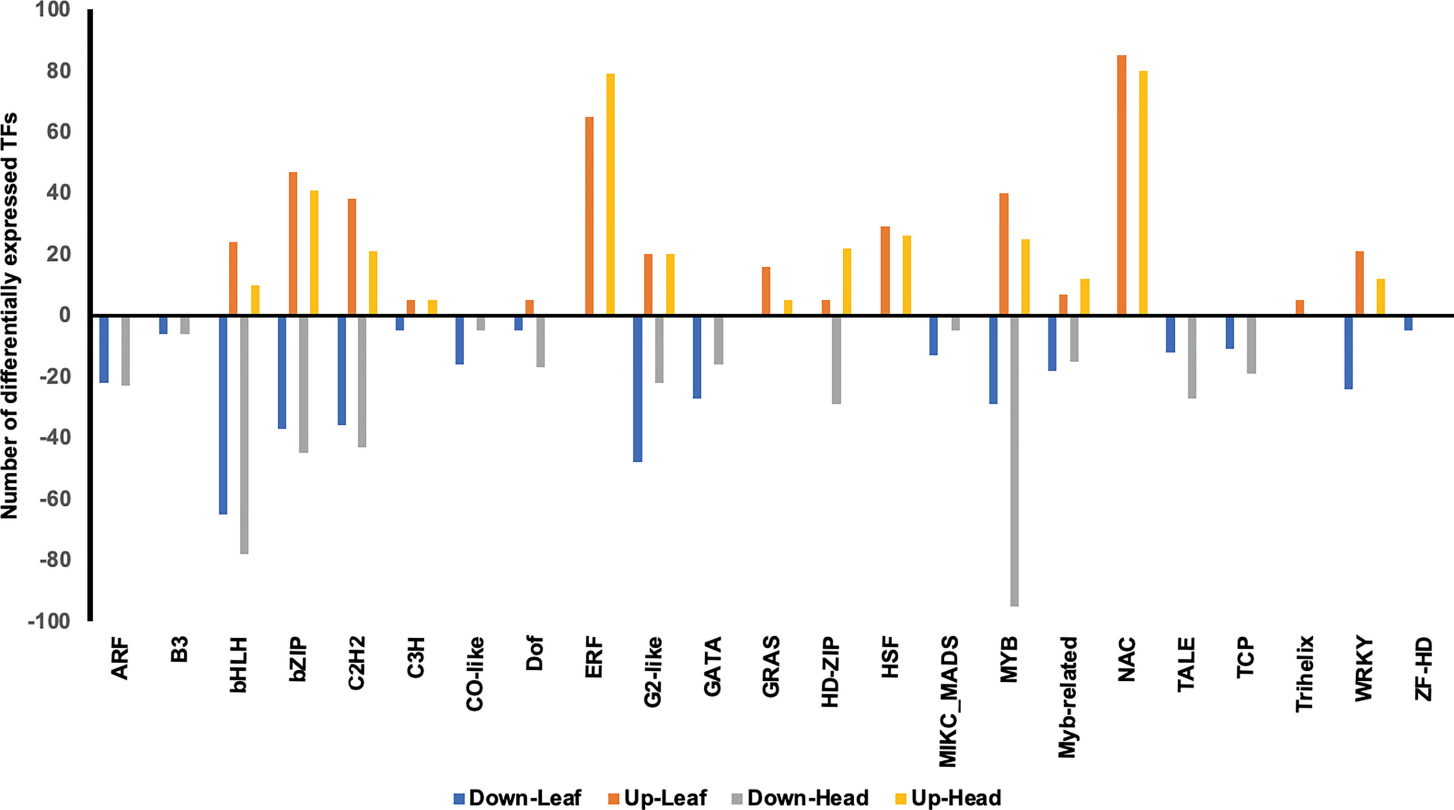
Transcription factors (TF) in leaf and head tissues that are differentially expressed in response to heat, drought and combined stress in Golden Promise and Otis. Only families of TFs with >5 expressed members are shown.

To get a better appreciation for the major transcription factor families associated with abiotic stress, an arbitrary cutoff of five or more members of a TF family was used as a selection criterion. This led to the identification of 23 TF families in the leaf tissues and 21 in the head tissues (**Figure 12** and **Supplementary Figures 4**-**7**). All the identified members of the ERF, GRAS, HD-Zip, HSF, NAC, and Trihelix family TFs were up regulated. All the identified members of ARF, B3, CO-like, GATA, MIKC-MADS, TALE, TCP and ZF-HD were down regulated. TFs belonging to the bHLH, bZIP, C2H2, C3H, Dof, G2-like, Myb, Myb-related and WRKY contained family members that were up or down regulated. The C3H family TFs were up in GP and down in Otis in response to combined stress (**Supplementary Figure 6**). In the head tissue the patterns of differential expression were like that observed in the leaf samples, except for few exceptions. The number of genes associated with the HD-Zip and Myb were substantially high in the heads compared to the leaf tissues.

### Innate differences in the transcriptomes of GP and Otis

Since GP is a malting variety and Otis is a feed barley, we set out to identify the innate differences in gene expression these two varieties in their leaf and head tissues. Since the tissue sampling was done at 1 and 5 days of stress treatments, control samples were also collected at the corresponding time points. For identifying genes differentially expressed between the two barley varieties, control samples were considered without respect to their timepoint (1-day, 5-day). Genes that were differentially expressed in two or more biological replicates were used for this analysis. In the flag leaf of GP 534 genes were differentially expressed (down in Otis) while 502 were identified in Otis (down in GP). In the head tissue of GP, 218 genes were differentially expressed compared to 238 in Otis (**Supplementary Table 9**).

KEGG pathway enrichment analysis was undertaken to identify the key differences that are associated with these two barley varieties. The comparisons between GP and Otis leaf samples identified three pathways that were reproducibly different between the biological replicates. The pathways were associated with glutathione metabolism, alpha-linolenic acid metabolism and starch-sucrose metabolism (**Table 1**). The glutathione pathway was also the lone pathway that was consistently differentially regulated in the head tissues.

**Table 1:** KEGG pathways enriched among differentially expressed genes in GP and Otis under non-stress conditions.

Gene Ids ^1^	GP:Otis^2^	Description
*Glutathione metabolism*		
1HG0021340	3.73	GST
1HG0023640	3.97	predicted protein
1HG0051740	3.56	glutathione S-transferase U17-like
1HG0051870	2.82	Tau GST
1HG0051910	4.80	glutathione S-transferase GSTU6
4HG0336870	1.75	glutathione S-transferase 3
2HG0169250	1.42	predicted protein
1HG0081150	*-2.13*	6-Phosphogluconate dehydrogenase
2HG0193390	*-1.71*	predicted protein
2HG0202910	*-1.17*	glutathione S-transferase GSTU6
2HG0214080	*-1.77*	glutathione peroxidase GPX15Hv
6HG0615590	*-1.47*	5-oxoprolinase
7HG0666960	*-1.64*	dehydroascorbate reductase
7HG0687590	*-1.59*	Ascorbate peroxidase
4HG0386180	*-1.60*	Glutathione-S-transferase
6HG0549380	*-1.42*	Gamma-glutamylcyclotransferase
*Starch and sugar metabolism*		
7HG0643810	1.94	nudix hydrolase 14
7HG0751660	2.18	starch branching enzyme
4HG0413910	2.61	Invertase, GH32 family
2HG0200340	3.51	Beta-fructofuranosidase
3HG0292350	2.20	Glycoside hydrolase family 3 GH3
5HG0478650	2.39	Endoglucanase
3HG0287930	*-2.38*	Hexokinase
7HG0735650	*-1.62*	Alpha-glucosidase
3HG0312760	*-2.31*	beta-glucosidase 5-like
7HG0712530	*-2.14*	1,4-alpha-glucan-branching enzyme
3HG0309930	*-3.15*	sucrose-phosphate synthase 1
5HG0525230	*-1.03*	adenylyltransferase
4HG0333180	*-2.17*	predicted protein
5HG0476550	*-1.87*	Trehalose-phosphatase
7HG0666870	*-1.27*	pfkB-like carbohydrate kinase
4HG0340370	*-1.90*	Glycoside hydrolase family 9
*Liniolenic acid metabolism*		
1HG0040000	2.46	3-ketoacyl-CoA thiolase 2
1HG0056730	1.39	Lipoxygenase
4HG0406770	2.95	alcohol dehydrogenase
2HG0170580	1.92	12-oxophytodienoic acid reductase
4HG0394970	*-3.49*	Allene oxide synthase
7HG0674860	*-1.41*	Acyl-CoA oxidase peroxisomal
5HG0420500	*-3.17*	Lipoxygenase
5HG0493180	*-1.90*	Alcohol dehydrogenase

^1^ Each of the gene identifiers should be preceded by HORVU.MOREX.R3.

^2^ The differential expression in GP:Otis non-stress plants. Regular font refers to genes that are up in GP while the negative italicized values represent genes that are up in Otis.

Glutathione metabolism: Of the 16 genes that were identified with this pathway in the leaves there were several interesting genes in ascorbate-glutathione pathway that were up in Otis compared to GP. This included key genes like the ascorbate peroxidase, dehydroascorbate reductase, glutathione peroxidase, gamma-glutamyl cyclotransferase and a predicted 5-oxoprolinase. It was interesting to note that a phosphogluconate dehydrogenase gene was up in Otis and could play a role in imparting stress tolerance by reducing oxidative stress. There were five GST genes that were upregulated in GP compared to Otis leaves. In the head tissue the most noteworthy genes identified as up in Otis were ascorbate peroxidase, ribonucleotide diphosphate reductase and spermidine synthase.

Alpha-linolenic acid metabolism: Eight genes were identified associated with this pathway. Interestingly, four genes were up in GP and four were up in Otis. Allene oxide synthase, lipoxygenase and Acyl-CoA oxidase are three genes identified in Otis that are associated with JA signaling. While 12-oxophytodienoic acid reductase 2, a lipoxygenase and an alcohol dehydrogenase were identified in GP.

Starch and sugar metabolism: Of the 16 genes associated with this pathway, 10 were up in Otis and six were up in GP. Among the genes that were up in Otis were a hexokinase, trehalose phosphatase, sucrose phosphate synthase, carbohydrate kinase, alpha and beta-glucosidase. In GP, a gene annotated as a starch branching enzyme, nudix hydrolase 14, invertase, and a glycoside hydrolase 3 family gene were identified.

## Discussion

In this study we chose to use the heat and drought stress tolerant Otis variety, a feed barley suited for the US-western drylands (with high temperatures and low moisture) and Golden Promise, a good malting variety, but sensitive to heat and drought stresses. Using model genotypes of *Hordeum vulgare* for identifying abiotic stress tolerance mechanisms will provide valuable genetic information for accelerating commercial cultivar development that can cater to the needs of the malting and brewing industries.

The physiological responses in both the varieties indicated a significant reduction in their stomatal conductance and transpiration rates in response to drought stress and higher than control levels in response to heat stress (**Figure 1**). Since the response to combined stress is like that of singly applied heat stress based on the increased stomatal conductance and transpiration rates it suggests that barley plants have innate abilities to withstand water deficits. However, based on the severe reduction in leaf water content, net photosynthesis rates and significantly higher seed yield loss observed in response to combined stress we speculate that barley plants perceive the combined heat and drought as an entirely different threat (**Figures 1**, **2**).

Pot-based experiments, such as the one described in this study, have the inherent disadvantage of not mimicking natural conditions, especially related to edaphic factors. On the contrary, experiments in controlled growth chamber settings aid in limiting variation due to interaction with environment. For example, rooting depth is not a trait for consideration, since the roots in these plants at heading stage readily explored all soil volume (although the pots were large). Thus, differences in soil exploring capacity (rooting depth) of these two varieties cannot be a factor for the genotypic disparities in physiological measurements. In fact, the root biomass data did not show any significant differences in the two varieties in response to heat or drought stress (**Figure 2**). Since the soil conditions and water availability were similar for the two genotypes, the more significant reduction in root biomass in response to combined stress in Otis compared to GP may be a stress tolerance strategy to divert the valuable resources for producing more seeds.

During terminal stresses such as heat and drought, shoot characteristics contribute to grain weight (Kobata et al., 1992; Sallam et al., 2014; Sallam et al., 2019). Drought or heat stresses during grain filling rapidly reduces photosynthesis which in turn reduces the available assimilates leading to significant reduction in kernel weight (Wardlaw and Willenbrink, 2000) (**Figure 2**). Therefore, reserves assimilated pre-anthesis is needed during grain filling (Gent, 1994). Shoot traits especially shoot dry weight has a strong linear relationship with the amount of carbohydrate remobilization under heat and drought stress (Kobata et al., 1992; Ehdaie et al., 2006; Xue et al., 2008). The higher shoot dry weight of GP observed in response to drought and heat stress suggests that poor remobilization of stem reserves could contribute to the lower seed yields. On the same lines, the significantly lower shoot dry weight in Otis in response to combined stress suggests efficient remobilization of stem reserves that contribute to the higher seed yields when compared to GP plants under stress (**Figure 2**).

A careful examination of the individual seeds shows significant differences between these two varieties. Otis is a two-row barley and in general has longer and plump seeds compared to GP which has much smaller grain size. The overall impact on seed length and width in response to drought was less pronounced compared to heat stress again indicating that the barley plants have innate ability to withstand water stress compared to temperature stresses (**Figure 3**). Duration of grain filling and the grain-filling rate are major contributors of grain plumpness. Reduction of nonstructural carbohydrates in the stems and vascular bundle impairment was associated with reduction in rice grain plumpness by heat stress during the early reproductive phase (Zhang et al., 2009; Wu et al., 2019). The significant reduction in both the seed length and width observed in GP compared to Otis in response to combined stress suggests grain filling processes such as synthesis and distribution of carbohydrates could be negatively impacted in the former. The increase in the seed length and the reduction in seed width in response to heat stress in GP is interesting and suggests changes associated with cell wall properties in this variety.

We chose QuantSeq method since it provides an easy protocol to generate strand-specific next-generation sequencing (NGS) libraries close to the 3’ end of polyadenylated RNAs. The main advantage of this strategy is that only one fragment per transcript is generated, directly linking the number of reads mapping to a gene to its expression. QuantSeq enables a higher level of multiplexing per run and provides accurate and affordable gene expression measurement. When compared with the normal RNA-seq wherein multiple reads can map to the same transcript thus tending to over-represent the longer transcripts, the 3’mRNA-seq method used in this study is set up to give one read per transcript and hence it is unbiased for transcript length. However, compared with normal RNA-seq libraries the total number of reads generated by this strategy is lower (**Supplementary Table 1**). The nearly 97% mapping of the reads from these libraries is significantly higher compared to other recent RNA-seq studies (Osthoff et al., 2019) wherein only 60% of the reads were mapped uniquely to the barley genome. We speculate that this improved mapping efficiency of the RNA-seq reads in this study was facilitated by the recently updated assembly of the barley genome (Morex V3) using the PacBio HiFi long read sequencing strategy (Mascher et al., 2021).

Among our three stress conditions and two timepoints, we found the least changes in gene expression at the end of 1-day of drought stress (**Figures 4**, **5**) similar to results reported on prolonged drought stress in a barley landrace (Cantalapiedra et al., 2017). Although related studies found more gene expression changes in leaves during early responses (Guo et al., 2009), plant responses to water deficit are different depending on genotype, phenology adjustment, acclimation, and developmental stage during which the stress is evaluated (Ashoub et al., 2015). Leaves from adult plants, like the ones in this study, will show different responses to drought than those of seedlings (Blum, 2005). Limited transcriptional response to drought stress in mature flowering plants could be due to acclimation or enhanced tolerance, maybe conferred by senescence of older leaves (Blum, 2009). No changes in leaf proteome of mature barley plants were reported under drought stress sampled three days into the treatment (Rollins et al., 2013). The importance of sampling tissues at different time-points is evident based on the significant changes in transcriptional responses that were observed in both genotypes at the end of the 5-day drought stress treatment (**Figures 5**, **6**).

Some processes that were found to be regulated in previous drought studies in barley were also identified in our analysis. This includes genes associated with carbohydrate metabolism, antioxidant enzymes like catalases, components of photosystem II (Krasensky and Jonak, 2012), proteases (Ford et al., 2011; Ashoub et al., 2013), lipoxygenases (Wendelboe-Nelson and Morris, 2012; Ashoub et al., 2015), and wax biosynthesis genes. Some common genes identified in drought studies in other plant species that came up in this study included transcription factors (TFs) from various families such as AP2/ERF, bZIP, DREB, NAC, WRKY (Sahoo et al., 2013; Janiak et al., 2016), genes associated with calcium signaling such as calcium sensor proteins, calmodulin, calcium-dependent protein kinases, and protein phosphatases class 2C (PP2C) (Molina et al., 2008; Guo et al., 2009; Ranjan and Sawant, 2015) and different members of the LEA family (Shinozaki and Yamaguchi-Shinozaki, 2007; Talame et al., 2007).

As reported in several other studies comparing barley genotypes with contrasting responses to drought (Rollins et al., 2013; Cantalapiedra et al., 2017; Harb et al., 2020), the sensitive variety in this study (GP) exhibited a higher number of differentially expressed genes than the tolerant one (**Figure 5**). However, some genes were found to be differentially expressed due to drought specifically in the relatively drought tolerant variety, Otis. Noteworthy genes included a calcium-dependent protein kinase, kynurenine formamidase, eceriferum 3, fatty acid hydroxylases, serine hydroxymethyltransferase, aspartate aminotransferase, sucrose synthase, sucrose symporter, several sugar transporters, 13 different genes associated with endopeptidase inhibitors that included two alpha-amylase inhibitors, four cysteine-type inhibitors, and seven serine-type endopeptidase inhibitors.

Biosynthesis of epicuticular waxes under stress conditions is mediated by *Eceriferum (CER)*s, an important gene family that plays key role in elongation of fatty acids chains (Ahmad et al., 2022). This gene was reported to be highly induced in Otis leaves in response to drought imposed during vegetative stage (Harb et al., 2020). In Arabidopsis overexpression of CER1 increased the very long chain alkenes in the cuticle and rendered the plants drought tolerant by preventing water loss from the leaves (Bourdenx et al., 2011). Elevated expression of two other genes annotated as fatty acid hydroxylase involved in wax biosynthesis in Otis may provide a mechanism for imparting the observed drought tolerance in this variety (**Figure 7**).

Proteases and Protease Inhibitors have been suggested to play a key role in adaptation to stress since it requires the active involvement of regulated proteolysis and the inhibition of uncontrolled proteolysis (Kidric et al., 2014). Lower proteolytic activity and decreased expression of certain cysteine protease genes under water deficit during early developmental stage are regarded as indicators for drought tolerance of winter wheat cultivars (Simova-Stoilova et al., 2010). Thus, the up regulation of cysteine protease inhibitors in Otis in response to drought could be involved in lowering the proteolytic activities (**Figure 7**).

The upregulation of four alpha-amylase inhibitor genes in Otis heads in response to drought is novel and exciting. It was reported that overexpression of wheat alpha-amylase inhibitor increased salt and drought tolerance (Xiao et al., 2013). It is known that, once amylase activity decreases (due to increased expression of alpha-amylase inhibitors), there is a concomitant reduction in the amount of glucose that is generated from starch breakdown. Subsequently, this lowers phosphoenolpyruvate (PEP) and oxaloacetate levels leading to a reduction in malic acid and cell turgor (Mansfield and Jones, 1971). This ultimately results in reduced stomatal apertures and/or its closure in leaves. However, the functional role of these inhibitors expressed in the heads needs further investigation.

### Heat Stress associated GOs

When compared to drought stress, heat stress evoked a larger transcriptional response by the end of the first day of treatment in both the genotypes. As a temperate cereal crop, it is not surprising that an increase in the temperature evoked substantial changes in the transcriptomes of these barley varieties. In contrast to drought stress, we identified enriched GOs wherein all the DEGs were identified in only one variety. For example, in Otis, there were seven carbonic anhydrase (CA) genes that were differentially expressed in response to heat of which two were up and five were down. Moderate stress, in general, leads to increased expression of CAs (Polishchuk, 2021). CAs plays a role in stress adaptation through its involvement in stomatal closure, ROS scavenging and partial compensation for reduced CO_2_ conductance in mesophyll. At high temperatures, CA gene expression was upregulated (Kaul et al., 2011), but its protein abundance and enzymatic activity were reportedly decreased (Ahsan et al., 2010; Chaki et al., 2013). Deactivation of CAs by high temperature (Boyd et al., 2015) could trigger a compensatory mechanism leading to their transcriptional upregulation. The association between CA and Rubisco enables interaction with CO_2_ and maintains the functional machinery of Rubisco (Badger and Price, 1994). Since photosynthesis is downregulated during severe stresses, suppression of CAs seems logical. Photosynthesis was reduced in both Otis and GP in response to heat stress, and it is still not clear why CAs are suppressed only in the former.

The enrichment of the GO for response to ABA in GP suggests higher levels of ABA. Of the 17 genes associated with this GO, 15 were differentially expressed only in GP and several of them have been shown to be induced in response to ABA. For example, HVA22 was initially identified as an ABA-induced transcript in barley aleurone layers (Shen et al., 1993). Another gene, Multiprotein Bridging Factor (MBF1), a key link that forms a bridge between stress responsive TFs and the basal transcription machinery (Jaimes-Miranda and Montes, 2020) has been reported to be induced by ABA (Yan et al., 2014) and heat in Arabidopsis (Suzuki et al., 2005), rice (Qin et al., 2015) and other plants. Upregulation of MBF1 in heads of GP agrees with the above-mentioned studies.

### Combined stress

In marked contrast to drought or heat stress, combination of drought and heat evoked a very extensive transcriptional reprogramming in both genotypes even within 24 hours after the treatment initiation. This clearly demonstrates that combined stress is not just an additive effect of heat and drought, but barley plants perceive this as a new threat. Combined heat and drought stress has been shown to evoke such massive transcriptional responses in other plants as well (Mahalingam, 2015; Mahalingam et al., 2021).

The GO term integral component of membrane contained the largest number of differentially expressed genes, exceeding more than 1000 in the sensitive GP leaves within 1-day of combined stress initiation and observed at the later time point. This GO is described as component of a membrane consisting of gene products with some part of their peptide sequence implanted in the hydrophobic region of the membrane. This suggests that changes in membrane composition and structure in GP could be a key factor for its susceptibility to combined heat and drought stress. Membrane damage is usually due to excess generation of reactive oxygen species (ROS) which attack lipid bilayers (Mahalingam and Fedoroff, 2003). Consistent with this hypothesis we identified that the GO for peroxisomes is significant in GP at both the early and later time points. Peroxisomes are ubiquitous subcellular organelles that are the major sites of ROS production in plants (del Rio and Lopez-Huertas, 2016). Several genes associated with H_2_O_2_ production in peroxisomes such as glycolate oxidase (GOX), amine oxidase, Acyl-CoA oxidases were unique to GP, or their expression was higher compared to Otis. It is important to note that the rate of H_2_O_2_ production by the peroxisomal GOX is several fold higher than that reported for chloroplasts and mitochondria (Foyer et al., 2009). Furthermore, superoxide generating enzymes such as uricase or urate oxidase and the superoxide dismutase were also differentially expressed suggesting a significant increase in the H_2_O_2_ production in response to combined stress. Several abiotic stresses are known to increase peroxisome abundance in Arabidopsis (Desai and Hu, 2008; Sinclair et al., 2009; Rodriguez-Serrano et al., 2016; Fahy et al., 2017), wheat (Sanad et al., 2019) and quinoa (Hinojosa et al., 2019). Peroxisomes were proposed as a cellular proxy for induction of ROS during stresses (Smertenko, 2017). In contrast to the above-mentioned studies where in stresses were imposed singly, the significantly lower peroxisome abundance observed in GP and Otis in response to combined stress merits scrutiny and this was also observed in quinoa in response to combined heat and drought stress (Hinojosa et al., 2019). In the light of the observation that several peroxins, genes associated with peroxisome proliferation such as PEX11, dynamin and PEX16 (Lingard et al., 2008) were higher in GP compared to Otis, it begs the question why the peroxisome abundance is significantly lower in GP compared to Otis in response to combined stress (**Figure 8**). Since upregulation of peroxisome proliferation genes did not reflect in higher peroxisome abundance under the combination of heat and drought, we speculate it may be offset by the higher rate of peroxisome degradation. Damaged peroxisomes (due to excess ROS) are eliminated by a process called pexophagy, a specialized type of autophagy (Farmer et al., 2013; Shibata et al., 2013). Higher autophagic flux could contribute to the reduced peroxisome abundance under the combination of drought and heat stress which is further supported by the enrichment of the GO for autophagy in the leaves of GP. Of the 19 genes associated with this GO, 11 were unique to GP and included several autophagy related genes such as ATG5, ATG9, ATG13, Beclin1, cysteine proteases (Shibata et al., 2013).

An interesting GO, cyclin-dependent protein ser/thr protein kinase inhibitor activity was identified in GP. One of the CDKI, was a Siamese-related protein that has been shown to restrict cell proliferation during leaf growth (Churchman et al., 2006). It has been shown in rice that these genes act as negative regulators of seed size and seed weight. CDKIs are also referred to as Kinase Inhibitor Proteins (KIPs) and Kip-related proteins (KRPs). *KRP1* overexpression transgenic lines (*OxKRP1*), *krp2* mutant *(crkrp2)*, and *krp1/krp2* double mutant *(crkrp1/krp2)* exhibited significantly reduced grain weight. Further, the seeds from these lines had seed germination issues and early seedling growth was retarded. This suggested that disturbing the normal steady state of KRP1 or KRP2 blocks seed development by impeding cell proliferation during grain filling and germination (Ajadi et al., 2020). Based on this rice study, it is tempting to speculate that the down regulation of the CDKIs (five in GP compared to two in Otis) could contribute to the reduced seed size in response to combined stress in GP.

Several genes associated with oxidoreductase activity that was induced early only in Otis in response to combined stress were very interesting and have documented roles in improving abiotic stresses. This includes genes involved in secondary metabolite production such as sorbitol, isoflavonoids, and atropine alkaloid, three ACC oxidase genes involved in the biosynthesis of phytohormone ethylene and two genes involved in the production of proline, a well-documented osmolyte (Al-Quraan and Al-Share, 2016). The upregulation of G3PDH gene in Otis is also consistent with a report on a wheat GAPDH gene that improved drought tolerance in a H_2_O_2_-mediated ABA-signaling pathway (Li et al., 2019).

WGCNA provides another strategy for identifying key genes important in response to abiotic stresses based on co-expression patterns that presumes interaction with other genes in the module (**Figure 9**). In gene networks, a subset of genes interacts with many other genes, and it is suggested that these hub genes are more likely to be essential than genes that have fewer interaction partners (Yokotani et al., 2008). It was interesting to find that four of the nine hub genes were associated with RNA metabolism including two different splicing factors, a polyA RNA polymerase and a RRM containing protein (**Figure 10**, **11**). Alternate splicing of key genes involved in abiotic stress such as HsfA2 in Arabidopsis (Liu et al., 2013), HvDRF1 in barley (Xue and Loveridge, 2004) and DREB in wheat (Egawa et al., 2006), maize (Qin et al., 2007) and tomato (Liu et al., 2017) have been reported. More than 300 genes were identified as being uniquely regulated by alternate splicing in response to drought in barley (Harb et al., 2020). Regulation of alternative splicing provides a mechanism to fine-tune gene expression that may save the time required for changes in transcriptional activation and pre-mRNA accumulation, thus facilitating rapid plant adaptation to adverse environmental conditions.

The removal of non-functional polypeptides due to aggregation, misfolding, denaturation is important for cellular homeostasis. Disassembly of heat shock-induced protein aggregates is aided by the Hsp100/ClpB (casein lytic protease) family proteins in conjunction with the Hsp70/Hsp40 system (Schirmer et al., 1996; Schilke et al., 2017). Heat shock induces the expression of plastid ClpB proteins in soybeans (Lee et al., 1994), lima bean (Keeler et al., 2000), and *A. thaliana* (Agarwal et al., 2001). Furthermore, the seedling-lethal phenotype exhibited by *clpb* mutants suggests an essential role for ClpB3 in normal development (Lee et al., 2007). The Hsp70 chaperone system is involved in folding and quality control of unfolded proteins (Schilke et al., 2017). It is a complex consisting of chaperone Hsp70 (DnaK), co-chaperone Hsp40 (DnaJ-type), and a nucleotide exchange factor (NEF). The binding and release of the substrate proteins are regulated by a cycle of ATP/ADP exchange. ATP hydrolysis on Hsp70 is accelerated by Hsp40 and substrate binding. Dissociation of ADP and rebinding of ATP causes the release of the bound substrate and this rate-limiting step in the ATPase cycle is regulated by the NEFs (Bukau and Horwich, 1998). In rice, Fes1, a NEF was found to directly interact with a salt responsive protein and Bip, an ER ortholog of HsP70 family and over/under-expression of Fes1 affected grain length and weight and improved salt tolerance (Qian et al., 2021). The Fes1 gene identified in barley is annotated as being localized to membranes and further functional analysis is warranted to confirm its role in abiotic stress tolerance.

### Transcription factors

The observed reprogramming of the transcriptome in response to heat, drought and combined stress involving nearly 10,000 genes in GP and more than 6000 genes in Otis is probably mediated by the differential expression of genes from 23 different transcription factor families (**Figure 12**).

Heat shock factors (HSFs) were identified in heat, drought, and combined stress in both genotypes. The expression of Heat-Shock-Proteins (HSPs) that function as chaperones to protect proteins under various stresses (Bartels and Sunkar, 2005) is regulated by HSFs (Rizhsky et al., 2004; Swindell et al., 2007; Guo et al., 2016). HSFs were more strongly induced in GP compared to Otis and the differential expression was highest in combined stress compared to drought or heat (**Supplementary Figures 4**, **6**). This type of modulation was also observed in Arabidopsis, wherein stronger induction of HSFA7B was reported in response to a combination of salt, osmotic and heat stress, when compared to its expression under heat stress (Sewelam et al., 2014). Thus, HSF and HSPs has been regarded as plausible targets for breeding or engineering plants with improved tolerance to abiotic stresses.

ERF TFs were another over-represented family that were identified in both single and combined stresses across time points (**Supplementary Figures 5**–**7** and **Supplementary Table 8**). In addition to their role in several developmental and physiological processes (Nakano et al., 2006) ERFs also act in response to wounding and in abiotic stresses (Mizoi et al., 2012; Heyman et al., 2018). Transgenic plants overexpressing certain ERFs are more resistant to several abiotic stresses including salinity, cold and water stress (Xu et al., 2008; Morran et al., 2011). In the present study, genes identified as ERF were both up and down-regulated (**Supplementary Figures 5**–**7**). DREB1A and DREB2A, two well studied ERFs were induced in rice in response to high salinity and water deficit (Dubouzet et al., 2003). In barley, the expression of DREB1A was significantly down-regulated in response to both short and long-term water deficit treatments while DREB2A was only slightly induced in long-term water deficit and combined salt and water-deficit stress (Osthoff et al., 2019). The precise functions of these barley ERFs need to be elucidated in future genetic analyses.

### Genes shaping stress tolerance in barley

Our study showed that the sensitive cultivar Golden Promise exhibited far more expression changes after combined heat and drought stress than the tolerant Otis genotype. This observation led to the hypothesis that Otis may have a “primed” transcriptome that is active under optimal conditions and hence does not need to instigate massive transcriptome changes when the stress occurs, as observed in the sensitive cultivar. This hypothesis was tested by the KEGG pathway enrichment analysis. The higher expression of key genes of the ascorbate-glutathione cycle in Otis can facilitate efficient ROS detoxification (Hasanuzzaman et al., 2019). Secondly, the innately higher expression of genes that are important for biosynthesis of osmolytes such as trehalose (Kosar et al., 2018) can provide a leg up for Otis in combating abiotic stresses. Thirdly the higher expression of key JA biosynthesis genes Allene oxide synthase, lipoxygenase and acyl-CoA oxidase could lead to higher levels of JA which has been shown to improve abiotic stress by enhancing antioxidants, osmolytes and cross-talk with other key phytohormones (Raza et al., 2021).

In addition to KEGG enrichment analysis of the innate differentially expressed genes we selected candidate genes based on two criteria (i) genes were differentially expressed only after application of stress in GP and (ii) the differential gene expression in GP in response to stress is like the expression pattern observed in Otis. It was surmised that such DEGs may be involved in better adaptation to stressful conditions.

Interestingly a gene annotated as a member of the universal stress response protein (USP) family (HORVU.MOREX.r3.6HG0618180) was identified in response to heat, drought, and combined stress. In plants USP functions include acting as protein and RNA chaperone, modulate ROS production, ABA-induced stomatal movement, Ethylene-mediated stress adaptation (Chi et al., 2019). In the barley genome there are 42 USPs and the identification of the USP in this study provides a strong rationale for its further functional characterization.

AP2/ERF gene family members in wheat were reported to show significant down regulation in response to heat and drought and one of the RAV subfamily members (TtAP2/ERF-117) contained a repressor motif (R/KLFGV) was down regulated in response to heat and drought stress (Faraji et al., 2020). The AP2/ERF gene (HORVU.MOREX.r3.1HG0083340) in barley had the same repressor motif and was down regulated in Otis under control conditions (compared with GP) and was repressed in response to heat and drought.

Homogentisate Phytyltransferase is a key enzyme for tocopherol biosynthesis (Collakova and DellaPenna, 2003). Tocopherols are well known lipid soluble antioxidants protecting cellular components from increased oxidative stress (Havaux et al., 2000; Munne-Bosch and Alegre, 2002). Identification of HPT as an innate gene with higher abundance in Otis adds one more important antioxidant metabolite that could aid in providing tolerance to abiotic stresses in this variety. In a previous study we have screened the wild barley diversity collection and the mini-core collection for all the eight isoforms of tocols (Mahalingam et al., 2020). Selecting lines with higher levels of tocopherols from these collections will provide additional novel barley lines for expanding the abiotic stress tolerance germplasm in barley.

Another novel transcription factor is the FAR1 (FAR-RED-Impaired Response 1), initially identified in Arabidopsis as crucial component of the phytochrome A-mediated far-red light signaling that has multifaceted roles in UV-B signaling, flowering, chloroplast biogenesis, ROS homeostasis, ABA signaling (Wang and Wang, 2015). Most of these studies have only been done in model systems and their functional studies in crops has not been reported. This study provides a strong rationale for pursuing functional characterization of this family of transcription factor in barley and is particularly fascinating given their extensive similarity to mutator-like transposase, indicative of molecular domestication (Hudson et al., 2003; Lin et al., 2007).

Starch is a key determinant of plant fitness especially during abiotic stresses (Thalmann and Santelia, 2017) since its remobilization can provide an alternate source of carbon which is limiting factor due to reduced photosynthesis. Alpha-amylases are important for starch breakdown in the leaves that can provide the much-needed energy supply and aid in the accumulation of compatible solutes. Increased amylase activity has been reported in response to abiotic stresses in rice, potato, Arabidopsis (Sicher, 2011; Valerio et al., 2011; Sitnicka and Orzechowski, 2014). Higher alpha-amylase activities in Otis may efficiently hydrolyze the transitory starch in the leaves to attenuate the impact of reduced photosynthesis in response to stress. This remobilization strategy could provide an alternate source of energy and carbon, promoting seed filling even during abiotic stress.

## Conclusions and future directions

Several well-known stress responsive genes and pathways associated with abiotic stress were found to be elevated in the feed barley variety Otis that could be conferring its tolerance to these stresses. Similar studies using commercial varieties (malting and feed barley) and advanced breeding lines with tolerance to abiotic stresses is warranted. Such pan-transcriptomics approach will enable identifying suites of stress tolerance genes that are innately expressed at higher levels. This information can be leveraged for selecting lines for breeding programs focused on developing malting and feed barley varieties with improved abiotic stress tolerance.

Engineering endogenous enzymes of barley could provide a direct approach to improving stress tolerance. For example, increased drought tolerance was reported in a CRISPR-generated Arabidopsis trehalase line wherein the edit mimicked the substrate binding site of an orthologous enzyme from the drought-tolerant *Selaginella lepidophylla* (Nunez-Munoz et al., 2021). We anticipate the innate stress responsive genes identified in Otis in conjunction with enzyme engineering offers an opportunity to accelerate success in producing climate-resilient barley varieties.

## Data availability statement

The data presented in the study are deposited in the SRA database, accession number PRJNA898434.

## Author contributions

RM and PB conceived the research project and approved the plan. PB provided the seeds and provided the fiscal support for the RNA-seq. RM, ND, and RK conducted the transcriptome analysis. AS and TN conducted peroxisome analysis and provided novel insights into the data analysis. RM wrote the manuscript with inputs from RK and AS. All authors contributed to the article and approved the submitted version.
