# Supplementary material for: Heat and drought induced transcriptomic changes in barley varieties with contrasting stress response phenotypes
Source: Front Plant Sci. 2022 Dec 8;13:1066421. doi: 10.3389/fpls.2022.1066421 (PMC9772561; doi:10.3389/fpls.2022.1066421)
Supplement: Supplementary file 1 [file Presentation_1.pptx]

## Slide 1
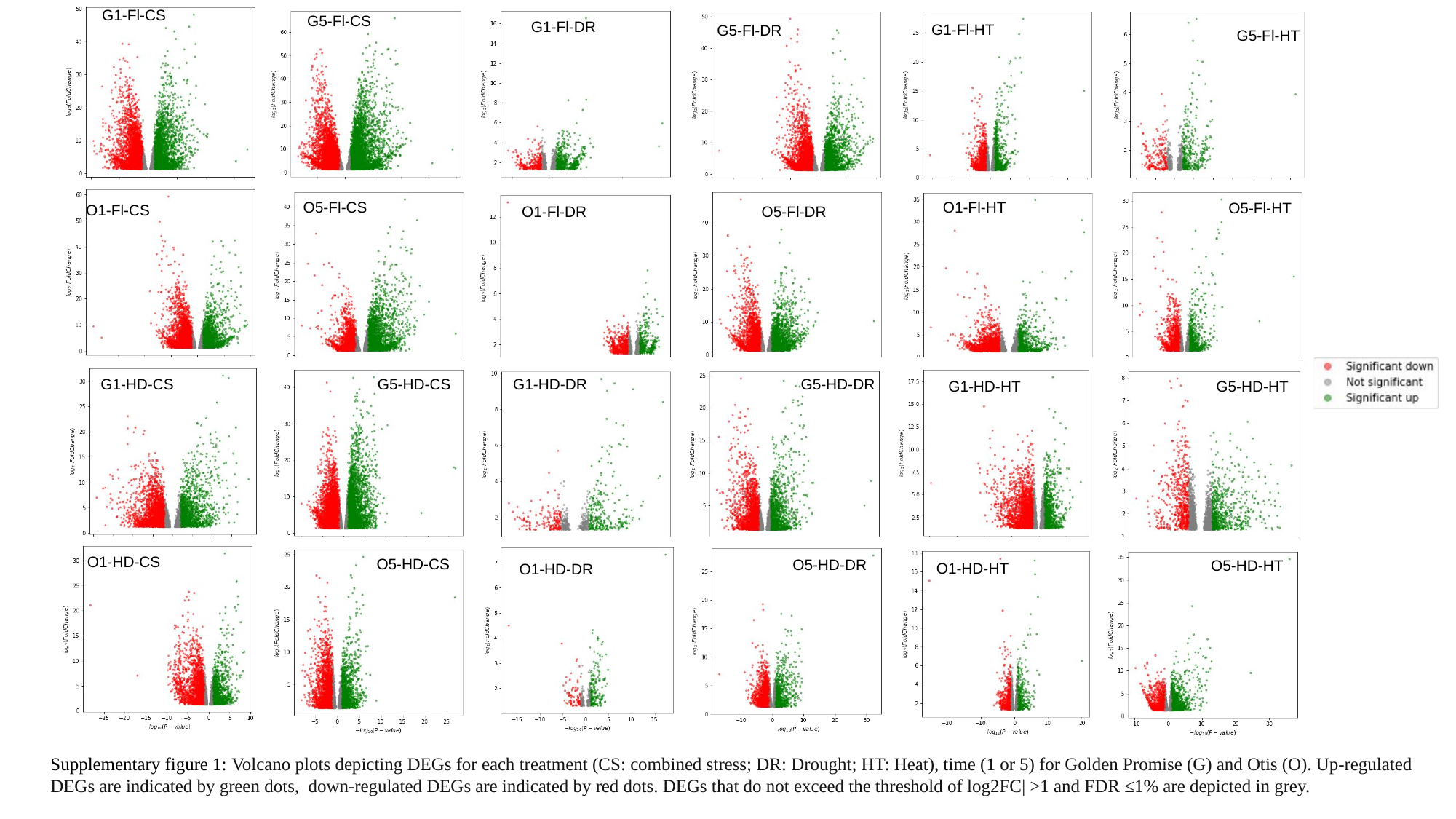

G1-Fl-CS
G5-Fl-CS
G1-Fl-DR
G1-Fl-HT
G5-Fl-DR
G5-Fl-HT
O1-Fl-HT
O5-Fl-CS
O5-Fl-HT
O1-Fl-CS
O5-Fl-DR
O1-Fl-DR
G5-HD-DR
G1-HD-CS
G5-HD-CS
G1-HD-DR
G1-HD-HT
G5-HD-HT
O1-HD-CS
O5-HD-CS
O5-HD-DR
O5-HD-HT
O1-HD-HT
O1-HD-DR
Supplementary figure 1: Volcano plots depicting DEGs for each treatment (CS: combined stress; DR: Drought; HT: Heat), time (1 or 5) for Golden Promise (G) and Otis (O). Up-regulated
DEGs are indicated by green dots, down-regulated DEGs are indicated by red dots. DEGs that do not exceed the threshold of log2FC| >1 and FDR ≤1% are depicted in grey.
