# Supplementary material for: Heat and drought induced transcriptomic changes in barley varieties with contrasting stress response phenotypes
Source: Front Plant Sci. 2022 Dec 8;13:1066421. doi: 10.3389/fpls.2022.1066421 (PMC9772561; doi:10.3389/fpls.2022.1066421)
Supplement: Supplementary file 2 [file Presentation_2.pptx]

## Slide 1
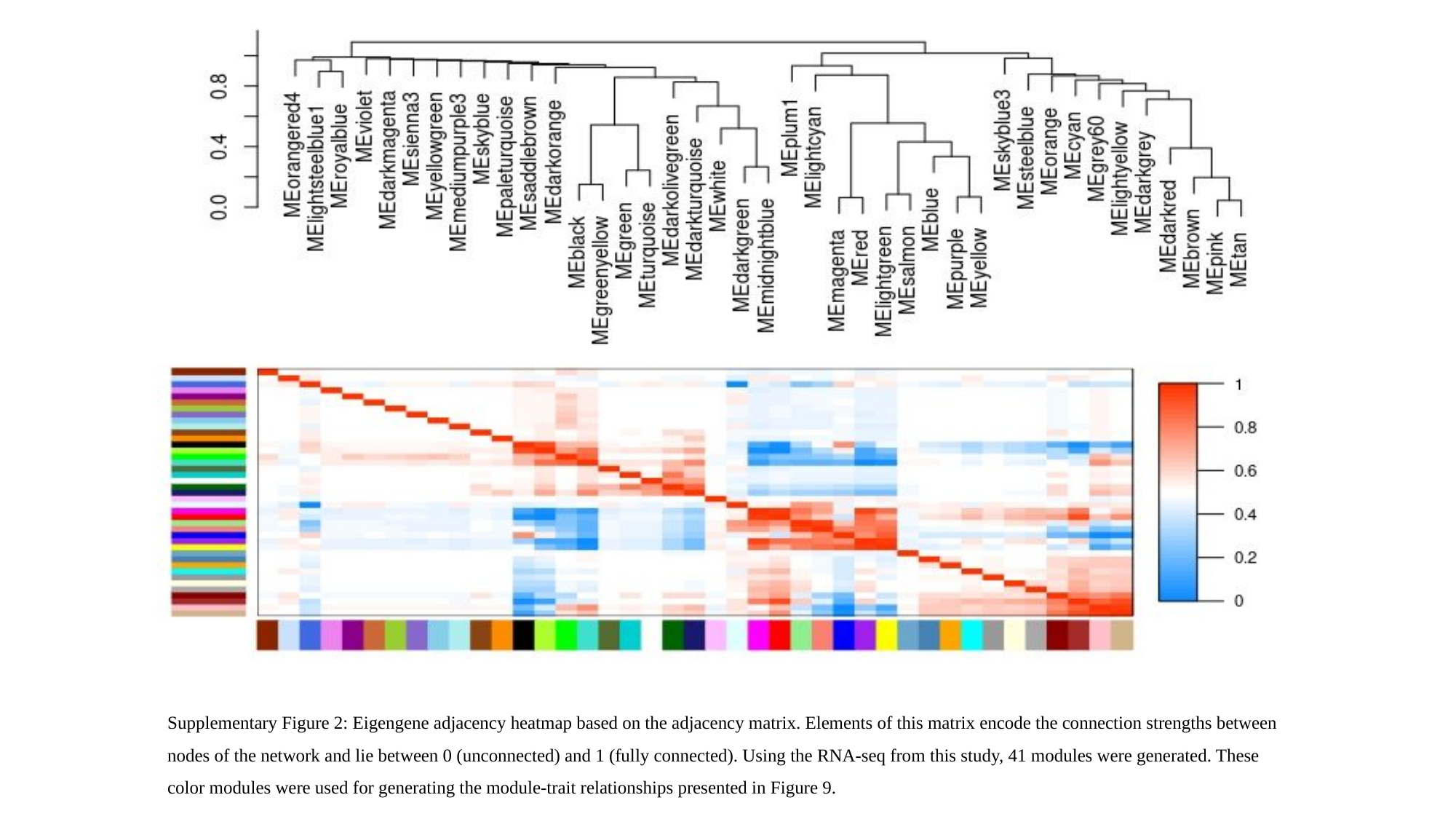

Supplementary Figure 2: Eigengene adjacency heatmap based on the adjacency matrix. Elements of this matrix encode the connection strengths between nodes of the network and lie between 0 (unconnected) and 1 (fully connected). Using the RNA-seq from this study, 41 modules were generated. These color modules were used for generating the module-trait relationships presented in Figure 9.
