# Supplementary material for: Heat and drought induced transcriptomic changes in barley varieties with contrasting stress response phenotypes
Source: Front Plant Sci. 2022 Dec 8;13:1066421. doi: 10.3389/fpls.2022.1066421 (PMC9772561; doi:10.3389/fpls.2022.1066421)
Supplement: Supplementary file 3 [file Presentation_3.pptx]

## Slide 1
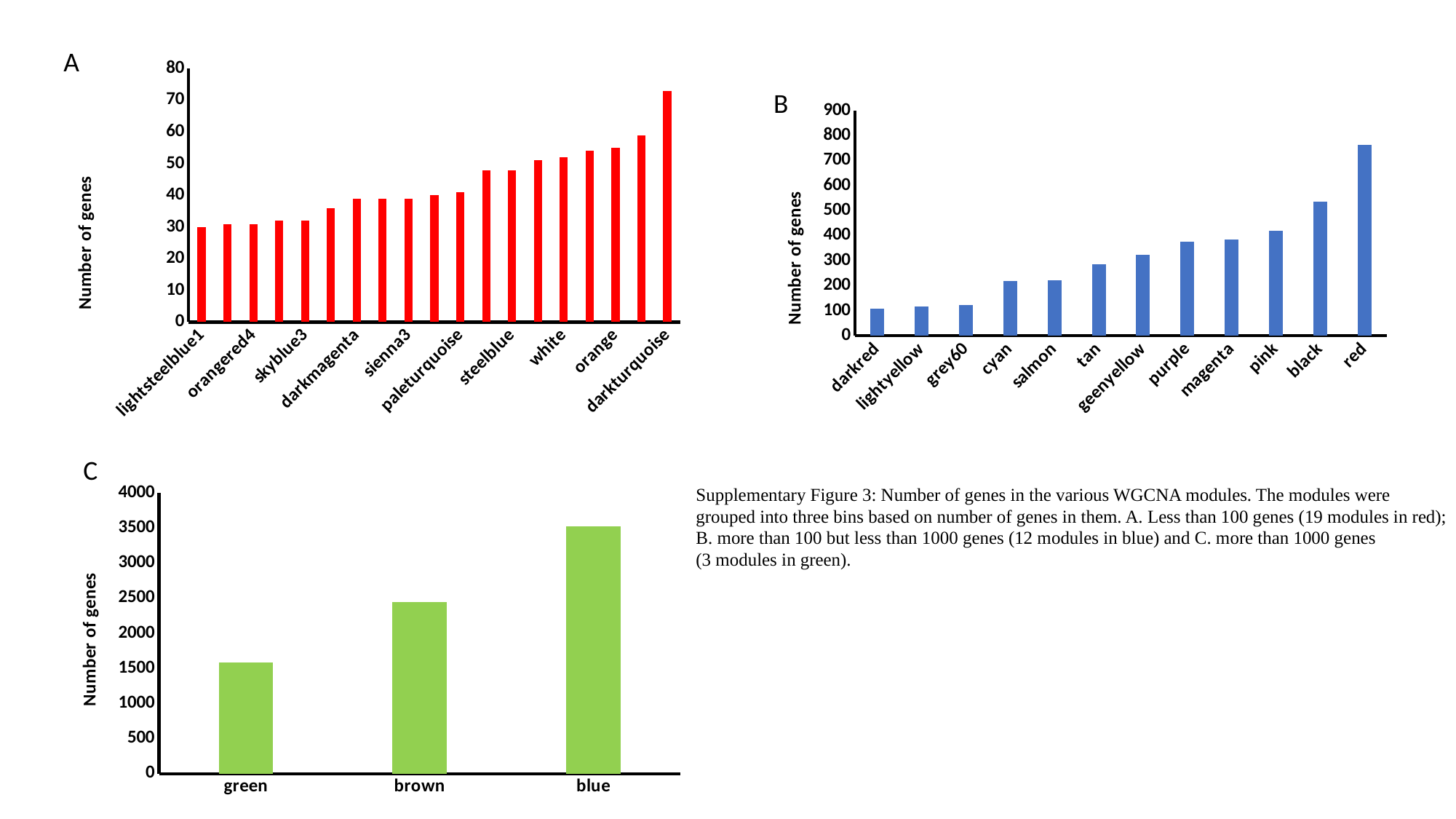

A
### Chart
| Category | Genes |
|---|---|
| lightsteelblue1 | 30.0 |
| mediumpurple3 | 31.0 |
| orangered4 | 31.0 |
| plum1 | 32.0 |
| skyblue3 | 32.0 |
| yellowgreen | 36.0 |
| darkmagenta | 39.0 |
| darkolivegreen | 39.0 |
| sienna3 | 39.0 |
| violtet | 40.0 |
| paleturquoise | 41.0 |
| saddlebrown | 48.0 |
| steelblue | 48.0 |
| skyblue | 51.0 |
| white | 52.0 |
| darkorange | 54.0 |
| orange | 55.0 |
| darkgrey | 59.0 |
| darkturquoise | 73.0 |B
### Chart
| Category | Genes |
|---|---|
| darkred | 108.0 |
| lightyellow | 117.0 |
| grey60 | 122.0 |
| cyan | 218.0 |
| salmon | 222.0 |
| tan | 287.0 |
| geenyellow | 325.0 |
| purple | 375.0 |
| magenta | 386.0 |
| pink | 420.0 |
| black | 535.0 |
| red | 763.0 |C
### Chart
| Category | Genes |
|---|---|
| green | 1582.0 |
| brown | 2443.0 |
| blue | 3527.0 |Supplementary Figure 3: Number of genes in the various WGCNA modules. The modules were
grouped into three bins based on number of genes in them. A. Less than 100 genes (19 modules in red);
B. more than 100 but less than 1000 genes (12 modules in blue) and C. more than 1000 genes
(3 modules in green).
