# Supplementary material for: Heat and drought induced transcriptomic changes in barley varieties with contrasting stress response phenotypes
Source: Front Plant Sci. 2022 Dec 8;13:1066421. doi: 10.3389/fpls.2022.1066421 (PMC9772561; doi:10.3389/fpls.2022.1066421)
Supplement: Supplementary file 4 [file Presentation_4.pptx]

## Slide 1
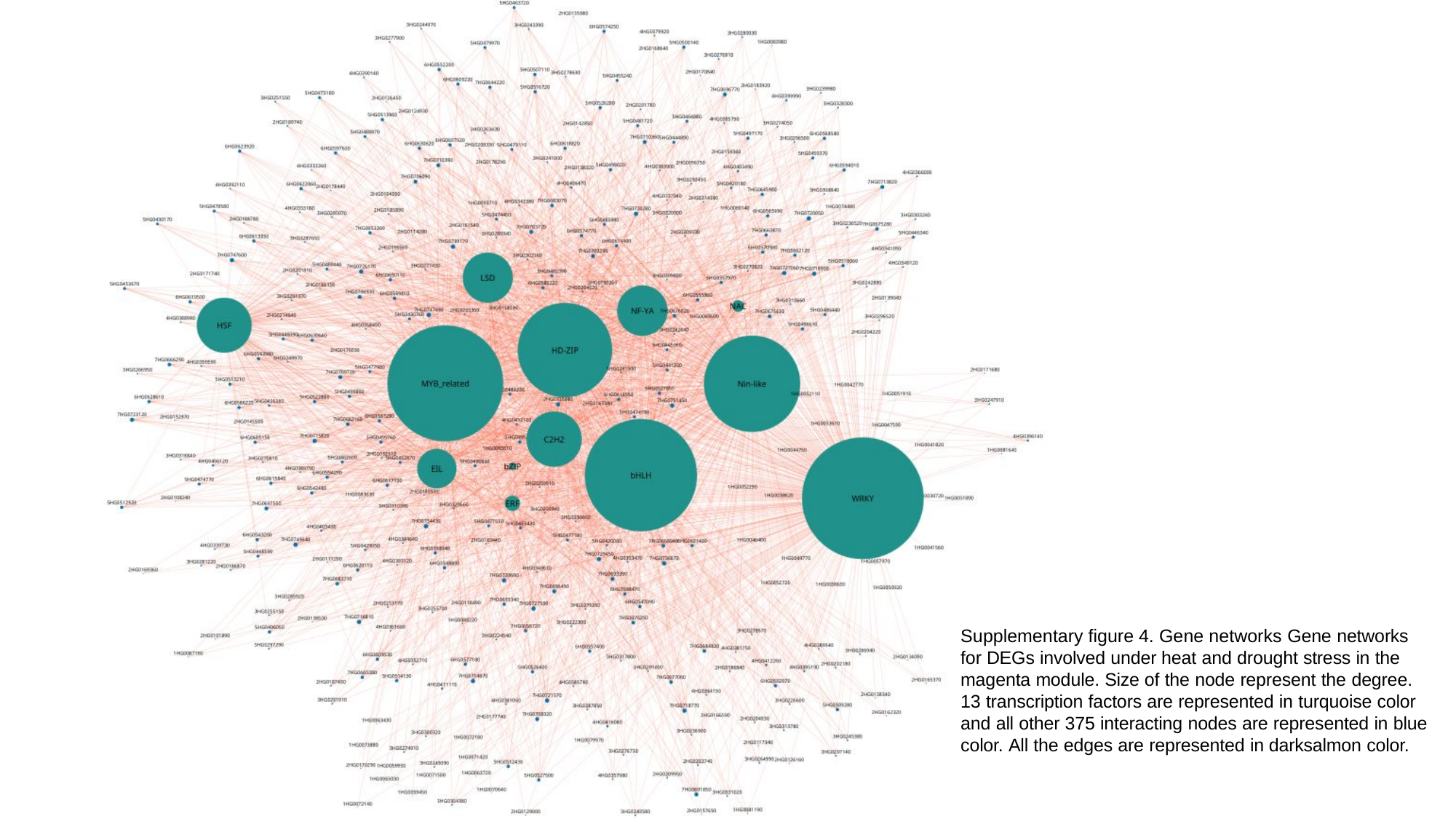

Supplementary figure 4. Gene networks Gene networks
for DEGs involved under heat and drought stress in the
magenta module. Size of the node represent the degree.
13 transcription factors are represented in turquoise color
and all other 375 interacting nodes are represented in blue
color. All the edges are represented in darksalmon color.
