# Supplementary material for: Heat and drought induced transcriptomic changes in barley varieties with contrasting stress response phenotypes
Source: Front Plant Sci. 2022 Dec 8;13:1066421. doi: 10.3389/fpls.2022.1066421 (PMC9772561; doi:10.3389/fpls.2022.1066421)
Supplement: Supplementary file 5 [file Presentation_5.pptx]

## Slide 1
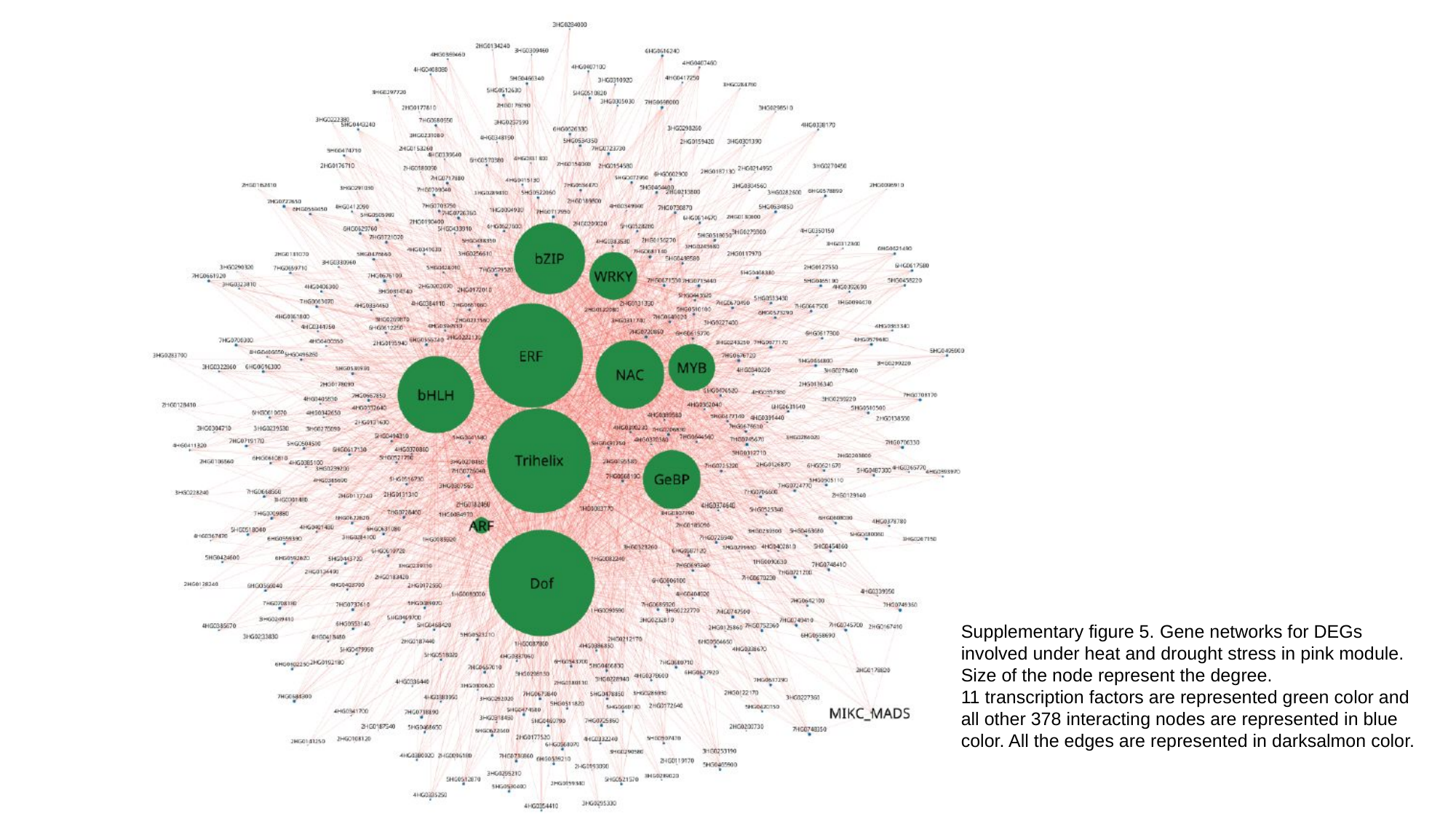

Supplementary figure 5. Gene networks for DEGs
involved under heat and drought stress in pink module.
Size of the node represent the degree.
11 transcription factors are represented green color and
all other 378 interacting nodes are represented in blue
color. All the edges are represented in darksalmon color.
