# Supplementary figures and images for: Heat and drought induced transcriptomic changes in barley varieties with contrasting stress response phenotypes
Source: Front Plant Sci. 2022 Dec 8;13:1066421. doi: 10.3389/fpls.2022.1066421 (PMC9772561; doi:10.3389/fpls.2022.1066421)

## Slide 1
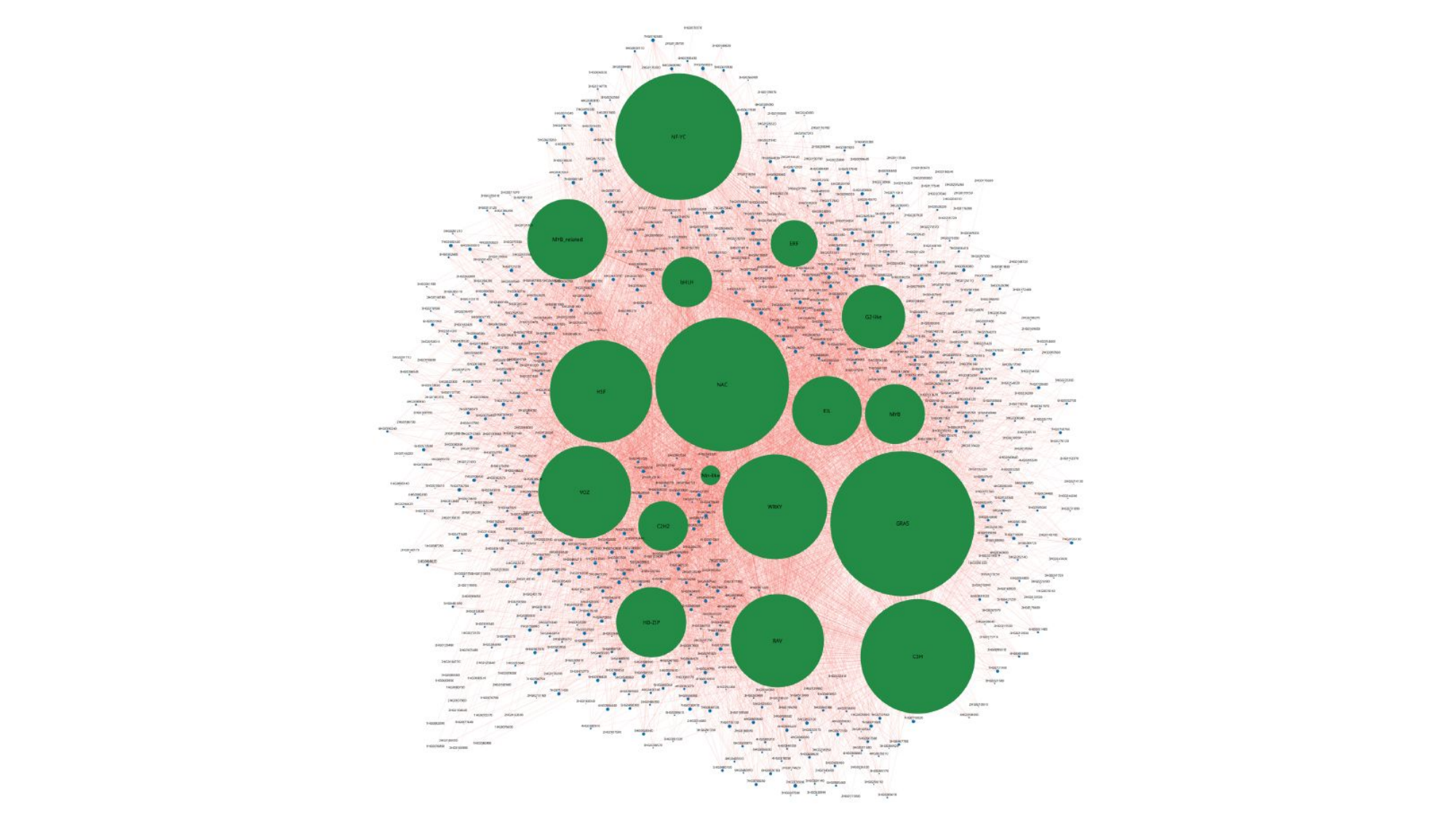

Supplement: Supplementary file 6 [file Presentation_6.pptx]
