# Supplementary material for: Heat and drought induced transcriptomic changes in barley varieties with contrasting stress response phenotypes
Source: Front Plant Sci. 2022 Dec 8;13:1066421. doi: 10.3389/fpls.2022.1066421 (PMC9772561; doi:10.3389/fpls.2022.1066421)
Supplement: Supplementary file 7 [file Presentation_7.pptx]

## Slide 1
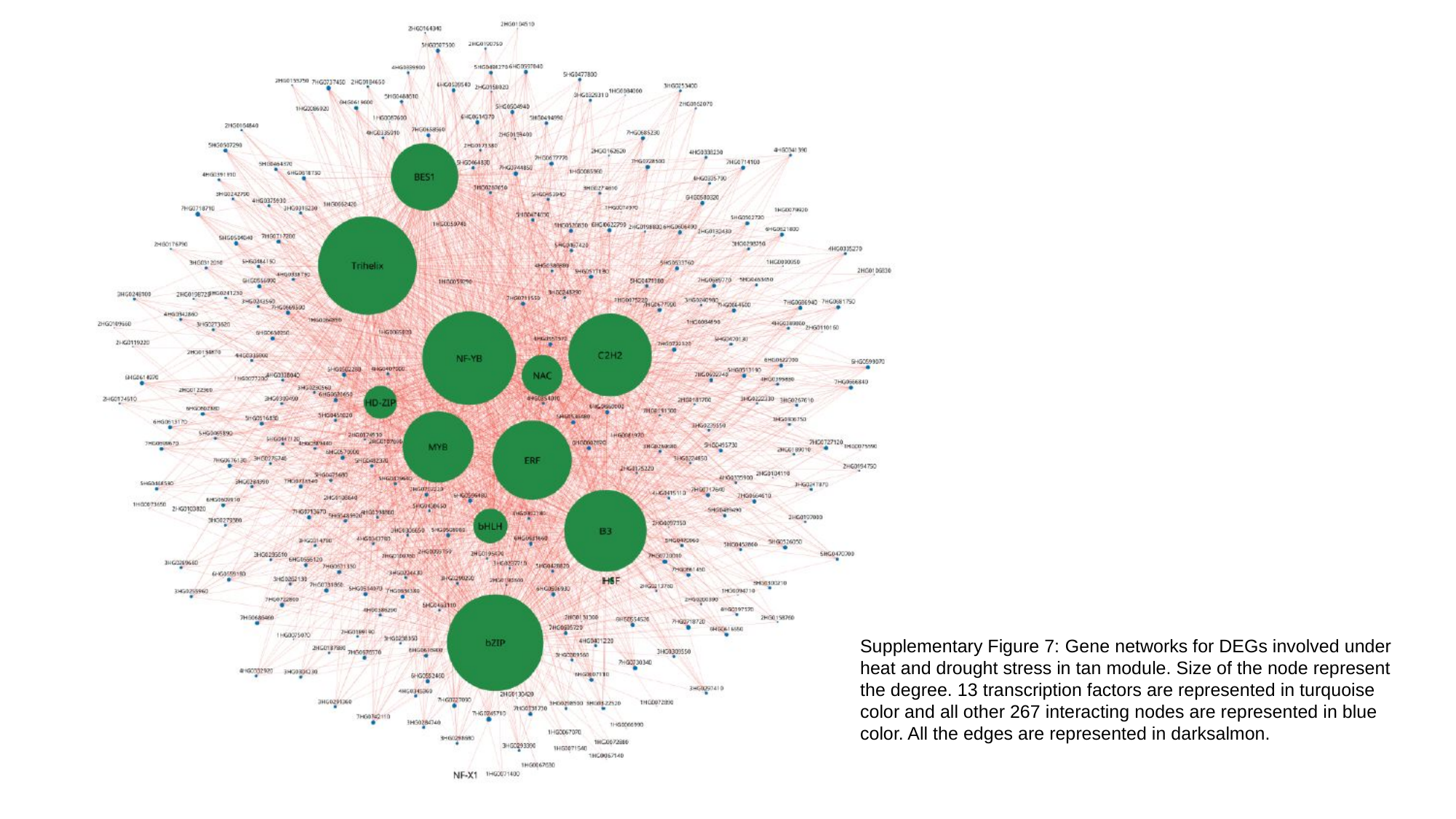

Supplementary Figure 7: Gene networks for DEGs involved under
heat and drought stress in tan module. Size of the node represent
the degree. 13 transcription factors are represented in turquoise
color and all other 267 interacting nodes are represented in blue
color. All the edges are represented in darksalmon.
